# Activation of Bivalent Gene POU4F1 Promotes and Maintains Basal‐like Breast Cancer

**DOI:** 10.1002/advs.202307660

**Published:** 2024-03-16

**Authors:** Jiahui Zhang, Nanyan Miao, Liyan Lao, Wen Deng, Jiawen Wang, Xiaofeng Zhu, Yongsheng Huang, Huayue Lin, Wenfeng Zeng, Wei Zhang, Luyuan Tan, Xiaoqing Yuan, Xin Zeng, Jingkun Zhu, Xueman Chen, Erwei Song, Linbin Yang, Yan Nie, Di Huang

**Affiliations:** ^1^ Guangdong Provincial Key Laboratory of Malignant Tumor Epigenetics and Gene Regulation Guangdong‐Hong Kong Joint Laboratory for RNA Medicine Breast Tumor Center Sun Yat‐sen Memorial Hospital Sun Yat‐sen University Guangzhou 510120 China; ^2^ Department of Plastic Surgery Sun Yat‐Sen Memorial Hospital Sun Yat‐Sen University Guangzhou 510120 China; ^3^ Center for Biotherapy Sun Yat‐sen Memorial Hospital Sun Yat‐sen University Guangzhou 510120 China; ^4^ Cellular & Molecular Diagnostics Center Sun Yat‐Sen Memorial Hospital Sun Yat‐Sen University Guangzhou 510120 China

**Keywords:** basal‐like breast cancer, bivalent chromatins, endocrine therapy, POU4F1, transcription factors

## Abstract

Basal‐like breast cancer (BLBC) is the most aggressive molecular subtype of breast cancer with worse prognosis and fewer treatment options. The underlying mechanisms upon BLBC transcriptional dysregulation and its upstream transcription factors (TFs) remain unclear. Here, among the hyperactive candidate TFs of BLBC identified by bioinformatic analysis, POU4F1 is uniquely upregulated in BLBC and is associated with poor prognosis. POU4F1 is necessary for the tumor growth and malignant phenotypes of BLBC through regulating G1/S transition by direct binding at the promoter of CDK2 and CCND1. More importantly, POU4F1 maintains BLBC identity by repressing ERα expression through CDK2‐mediated EZH2 phosphorylation and subsequent H3K27me3 modification in *ESR1* promoter. Knocking out POU4F1 in BLBC cells reactivates functional ERα expression, rendering BLBC sensitive to tamoxifen treatment. In‐depth epigenetic analysis reveals that the subtype‐specific re‐configuration and activation of the bivalent chromatin in the *POU4F1* promoter contributes to its unique expression in BLBC, which is maintained by DNA demethylase TET1. Together, these results reveal a subtype‐specific epigenetically activated TF with critical role in promoting and maintaining BLBC, suggesting that POU4F1 is a potential therapeutic target for BLBC.

## Graphical abstract

The bivalent gene POU4F1 is epigenetically activated in basal‐like breast cancer by DNA demethylase TET1. POU4F1 promotes cell cycle progression through binding in the promoter of *CDK2* and *CCND1*. CDK2 further phosphorylates EZH2, leading to H3K27me3 modification and repressed expression of ERα. Targeting POU4F1 not only inhibits tumor growth, but also conferring anti‐estrogen sensitivity.

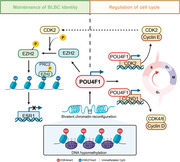

## 1 Introduction

Breast cancer is the most commonly diagnosed cancer worldwide, having overtaken lung cancer for the first time in 2020.^[^ ^1^ ^]^ Basal‐like breast cancer (BLBC), accounting for ≈15%–20% of breast cancer, is classified based on the transcriptional program of breast cancer with a characteristic of high proliferation activity and cell‐cycle checkpoint dysregulation.^[^ ^2^ ^]^ Approximately 75% of BLBCs are triple negative breast cancers (TNBCs), which do not express estrogen receptor (ER), progesterone receptor (PR) and human epidermal growth factor receptor 2 (HER2).^[^ ^3^ ^]^ Therefore, endocrine therapy and HER2‐targeted therapy are ineffective, with surgery and chemotherapy remaining the major therapeutic treatments.^[^ ^4^ ^]^ The inherently aggressive clinical behavior, coupled with the lack of effective molecular targets, result in the poor prognosis of BLBC.^[^ ^3^, ^4^ ^]^ Despite substantial genetic information available for BLBC, no clear genetic oncogenic drivers have been identified for BLBC.^[^ ^5^ ^]^ Therefore, exploring the mechanisms underlying the tumorigenesis and progression of BLBC is essential to discover new therapeutic targets for BLBC.

Transcriptional dysregulation is a hallmark of cancer and can be essential drivers for cancer onset and progression.^[^ ^6^ ^]^ A growing number of studies have confirmed that dysregulated transcriptional program can be dominated by transcription factors (TFs), which are key proteins involved in the regulation of gene transcription.^[^ ^7^ ^]^ A limited list of TFs is overactive in specific cancer types and represents a unique class of drug targets.^[^ ^8^ ^]^ For example, ERα is one of the master TFs in regulating luminal phenotype of breast cancer and the key target of endocrine therapy.^[^ ^9^ ^]^ However, the master TFs and the underlying regulatory mechanisms in BLBC remained unclear.

TFs specifically bind to DNA sequence of the promoters and enhancers in genome. Increasing studies indicated that the TF binding‐enhancer region was dramatically altered in DNA methylation profiles and strongly correlated to cell type‐specific gene expression.^[^ ^10^ ^]^ In breast cancer, aberrant DNA methylation in enhancer regions has been shown to be associated with different molecular subtypes.^[^ ^10^, ^11^ ^]^ Therefore, cancer‐specific TFs can be inferred from DNA methylation profiles of enhancer regions containing putative TF binding sites. In this study, we aimed to investigate the BLBC‐specific TFs in regulating tumor progression, with the goal of exploring new therapeutic strategies to target BLBC.

## 2 Results

### 2.1 POU4F1 is Specifically Upregulated in BLBC and Associated with Worse Prognosis

**Figure 1:**
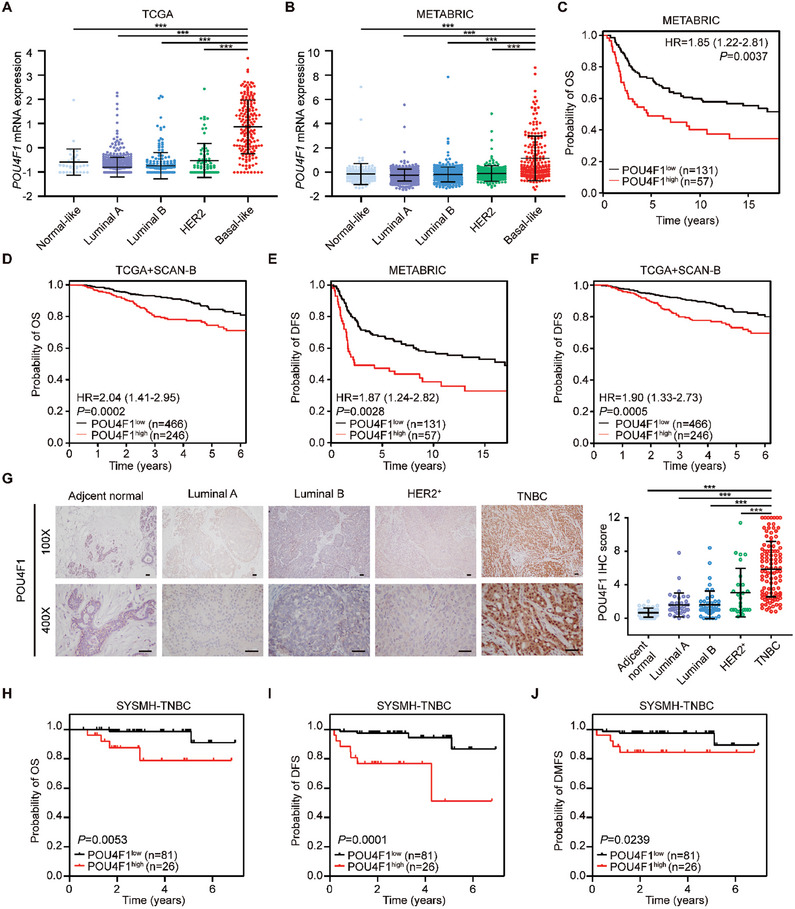
POU4F1 is uniquely upregulated in BLBC and is associated with poor prognosis. A,B) *POU4F1* mRNA expression across PAM50 subtypes of breast cancer patients in the TCGA (A) (Basal‐like: *n* = 171, HER2‐enriched: *n* = 78, Luminal A: *n* = 499, Luminal B: *n* = 197, Normal‐like: *n* = 36) and METABRIC (B) (Basal‐like: *n* = 199, HER2‐enriched: *n* = 220, Luminal A: *n* = 679, Luminal B: *n* = 461, Normal‐like: *n* = 140) cohorts. C–F) Kaplan‐Meier survival plots showing overall survival C,D) and disease‐free survival E,F) of BLBC patients in the METABRIC C,E) and the TCGA and SCAN‐B D,F) cohorts with high or low POU4F1 expression. *P* value was determined by univariate cox regression analysis. Hazard ratio (HR) and 95% confidence interval (95% CI) are shown. G) Representative IHC images and immunoreactive scores (IRS) of POU4F1 expression in different subtypes of breast cancer and adjacent normal breast (TNBC: *n* = 107, Luminal A: *n* = 38, Luminal B: *n* = 46, HER2+: *n* = 27, adjacent normal breast: *n* = 40). Scale bar, 50 µm. H–J) Kaplan‐Meier survival plots showing overall survival H), disease‐free survival I) and distant metastasis‐free survival J) of TNBC patients with high or low IHC score of POU4F1 (n = 107). Log rank P were shown. Data were presented as mean ± S.D. A,B,G). ***P < 0.001 compared with indicated subtypes by two‐tailed one‐way ANOVA and Dunnett's multiple‐comparisons test A,B,G).

To identify BLBC‐specific hyperactive TFs, Enhancer Linking by Methylation/Expression Relationships (ELMER), an available computational method used to identify master TFs of specific cancer subtypes,^[^ ^12^ ^]^ was employed to identify the differentially methylated TF‐binding enhancer regions and corresponding TFs by using DNA methylation and RNA sequencing (RNA‐seq) data in the TCGA database (Figure S1A, Supporting Information). In this way, 15 hyperactive TFs have been identified as candidate master TFs of BLBC and performed the following screening strategies (Figure S1A, Supporting Information). First, since cancer‐specific TFs are generally expressed in cell type‐specific manner, we compared the mRNA expression of these candidate TFs in different breast cancer subtypes in the TCGA and METABRIC cohorts. Except *SOX11*, *KLF5*, *LHX2*, *MYBL2*, *ZIC1*, *TLX3* and *NKX1‐2*, 8 TFs including *POU4F1*, *NFE2L3*, *EN1*, *FOXC1*, *ETV6*, *BCL11A*, *CEBPB*, *ZNF232*, showed BLBC‐specific expression in the TCGA and METABRIC cohorts (**Figure** **1**A,B; Figure S1B,C, Supporting Information). Next, Kaplan‐meier curves and univariate Cox regression analysis were performed to screen the candidate TFs that correlated with the survival of BLBC patients. Among these 8 candidates, high levels of *EN1* and *POU4F1* were significantly associated with short overall survival (OS) of BLBC patients in the METABRIC cohort, TCGA and Sweden Cancerome Analysis Network‐Breast (SCAN‐B) cohorts (Figure 1C,D; Figure S1D,E, Supporting Information). However, only POU4F1 specifically provided prognostic value in BLBC but no other subtypes (Figure S1E,F, Supporting Information). Additionally, tumor with higher expression of POU4F1 showed shorter disease‐free survival (DFS) in BLBC patients in the METABRIC, TCGA and SCAN‐B cohorts (Figure 1E,F).

Consistent with the online TCGA and METABRIC database, POU4F1 expression, determined by immunohistochemistry staining, was significantly higher in breast cancer tissues of TNBC patients, compared with ones of luminal A, luminal B and HER2‐positive breast cancer patients and normal breast tissues (Figure 1G). Similarly, higher expression of POU4F1 was associated with shorter OS, DFS and distant metastasis‐free survival (DMFS) in TNBC patients (Figure 1H–J). Furthermore, univariate and multivariate Cox regression analyses revealed that POU4F1 expression was an independent risk factor for TNBC patients (Tables S1 and S2, Supporting Information). Taken together, we validated that POU4F1 was specifically upregulated in BLBC patient samples and associated with poor prognosis from external and internal datasets.

### 2.2 POU4F1 is Required for the Malignant Phenotypes of BLBC

**Figure 2:**
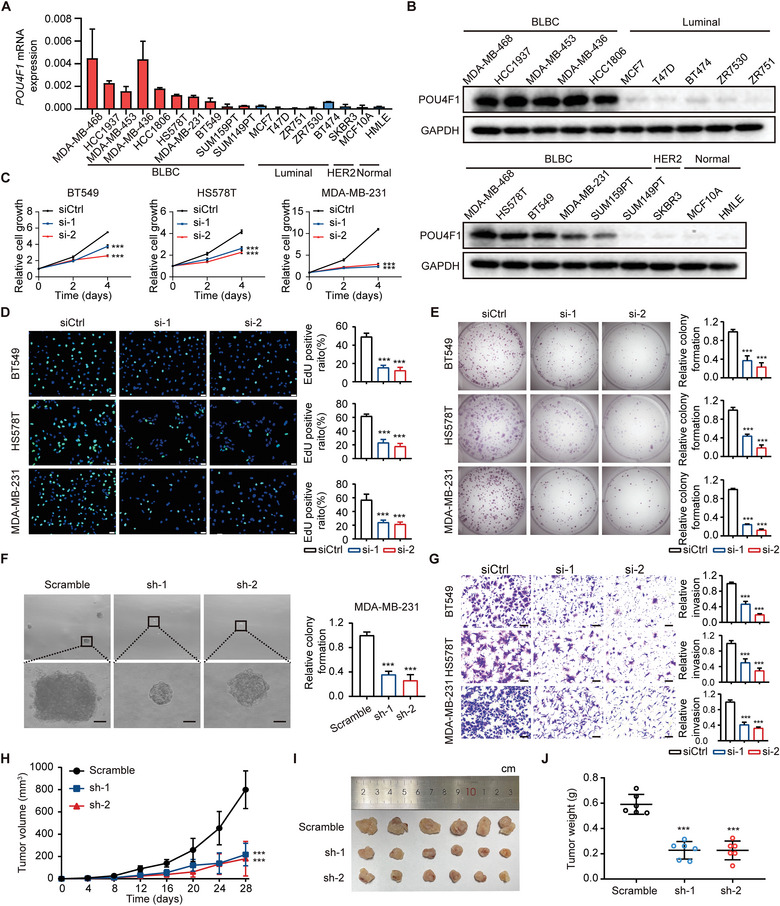
POU4F1 is required for the malignant phenotypes of BLBC. A,B) The expression of *POU4F1* in breast cancer cell lines and normal breast epithelial cell lines, measured by qRT‐PCR A) and western blotting B). C,D) Cell proliferation of 3 BLBC cell lines (BT549, HS578T, MDA‐MB‐231) transfected with POU4F1 siRNAs and control siRNA, detected by CCK8 cell proliferation assay C) and 5‐Ethynyl‐2'‐deoxyuridine (EdU) assay D). Scale bar, 50 µm. E) Representative images and quantitation of the colony formation of 3 BLBC cell lines transfected with POU4F1 siRNAs and control siRNA. F) Representative images and quantitation of the anchorage‐independent growth assays in soft agar of MDA‐MB‐231 cells after shRNA‐mediated knockdown of POU4F1. Scale bar, 50 µm. G) Representative images and quantitation of cell invasion of 3 BLBC cell lines transfected with POU4F1 siRNAs and control siRNA, assessed through Boyden chambers assay. Scale bar, 50 µm. H–J) Tumor growth curves H), tumor images I) and tumor weight on day28 J) of xenografts of MDA‐MB‐231 cells transduced with shRNA targeting POU4F1 or scramble. Data were presented as mean ± S.D. A,C–H,J), *n* = 3 A,C–G) and *n* = 6 H–J). ***P < 0.001 compared with siCtrl C–E,G) and scramble F,H,J) by two‐tailed one‐way ANOVA and Dunnett's multiple‐comparisons test.

To investigate the biological function of POU4F1 in BLBC, we first validated the uniquely high expression of POU4F1 in BLBC cell lines by qRT‐PCR and western blotting, which was consistent with the RNA‐seq or microarray data in another three independent studies, including Heiser et al, Neve et al and the Cancer Cell Line Encyclopedia (CCLE) project (**Figure** **2**A,B; Figure S2A, Supporting Information).^[^ ^13^ ^]^ Then we silenced POU4F1 by two independent small interfering RNAs (siRNAs) in BLBC cell lines and validated the silencing efficiency using qRT‐PCR and western blotting (Figure S2B,C, Supporting Information). Determined by CCK8 cell proliferation assay and 5‐Ethynyl‐2´‐deoxyuridine (EdU) assay, silencing POU4F1 significantly inhibited cell growth in BLBC cell lines (Figure 2C,D; Figure S2D,E, Supporting Information). Moreover, silencing POU4F1 markedly diminished the colony formation capacity of BLBC cell lines in both colony formation and anchorage‐independent growth assays, as well as inhibited the migration and invasion of BLBC cell lines, but not influenced apoptosis in BLBC cell lines (Figure 2E–G; Figure S2F–K, Supporting Information). Consistently, POU4F1 overexpression enhanced the migration and invasion of BLBC cell lines (Figure S3A–C, Supporting Information).

To further validate the function of POU4F1 in vivo, MDA‐MB‐231 cells stably transduced with shRNAs targeting POU4F1 or non‐targeting control shRNA were inoculated into the fatpads of nude mice, respectively. Silencing POU4F1 significantly inhibited tumor growth， reduced the percentages of Ki67‐positive cancer cells and decreased lung metastasis (Figure 2H–J; (Figure S4A–C, Supporting Information). Taken together, these results demonstrated that POU4F1 was required for the tumor growth and the malignant phenotypes of BLBC, including proliferation, colony formation, migration and invasion.

### 2.3 POU4F1 Drives the Malignant Progression of BLBC via Upregulating Cell‐Cycle Related Pathway

**Figure 3:**
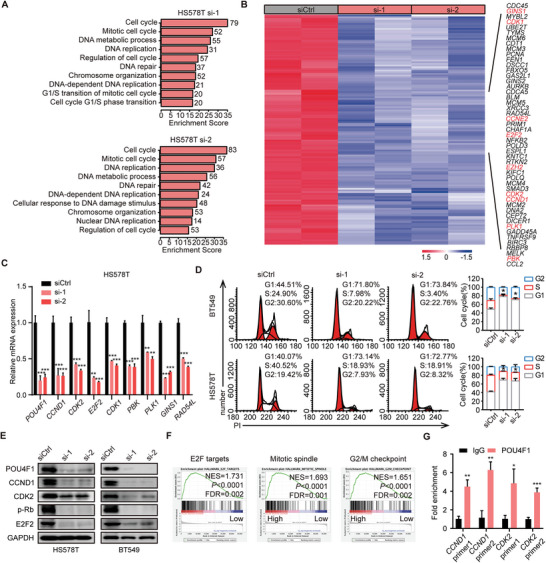
POU4F1 regulates cell cycle progression in BLBC. A) Top 10 GO categories of the differentially downregulated genes (fold change > 1.5 and *P* < 0.05) in HS578T transfected with POU4F1 siRNAs and control siRNA, as measured by RNA‐seq with two biological duplicates. B) Heatmap of the overlap differentially downregulated genes of two siRNA targeting POU4F1 in HS578T cells. C) qRT‐PCR detection of the expression of the representative cell cycle‐related genes in HS578T transfected with POU4F1 siRNAs and control siRNA. D) Representative flow cytometry histograms and quantification of cell cycle distribution in BT549 and HS578T transfected with POU4F1 siRNAs and control siRNA. E) Western blotting showing the expression of indicated proteins in BT549 and HS578T transfected with POU4F1 siRNAs and control siRNA. F) The top three significant gene sets enriched in patients with high *POU4F1* expression, identified by GSEA of BLBC patients in TCGA cohorts. The patient cohort was divided into the top 30% and bottom 30% based on *POU4F1* mRNA expression. G) ChIP‐qPCR analysis of the enrichment of POU4F1 at the promoter of *CCND1* and *CDK2*. Data were presented as mean ± S.D., *n* = 3 C, D, G). *P < 0.05, **P < 0.01, ***P < 0.001 compared with siCtrl C,D) or IgG G) by two‐tailed one‐way ANOVA and Dunnett's multiple‐comparisons test C,D) and two‐tailed Student's t‐test G).

To investigate how POU4F1 regulates BLBC progression, we performed RNA‐seq on two BLBC cell lines transfected with POU4F1 siRNAs and analyzed differentially expressed genes (fold change > 1.5 and *P* < 0.05). Gene ontology (GO) analysis of the differentially downregulated genes showed that the top 10 GO categories were associated with cell cycle (**Figure** **3**A; Figure S5A, Supporting Information). Consistently, Kyoto encyclopedia of genes and genomes (KEGG) analysis also showed that cell cycle was the most significant enriched pathway of the differentially downregulated genes (Table S3, Supporting Information). The expression of representative genes regulating cell cycle, including *E2F2, CCND1, CDK1, CDK2, PBK, PLK1, GINS1* and *RAD54L*, were further validated by qRT‐PCR, showing that these genes were decreased in BLBC cells with POU4F1 knockdown and increased in BLBC cells with POU4F1 overexpression (Figure 3B,C; Figure S5, Supporting Information). We then assessed the cell cycle profiles by flow cytometry and observed significant decrease in S phase and a corresponding increase in G1‐phase upon POU4F1 knockdown, which indicated G1/S phase arrest (Figure 3D). In contrast, POU4F1 overexpression resulted in a significant increase in S phase (Figure S5F, Supporting Information). The key genes involved in G1/S phase transition, including CCND1, CDK2 and downstream phosphorylated‐Rb and E2F2 were both significantly downregulated in HS578T and BT549 cells with POU4F1 knockdown, as determined by western blotting (Figure 3E). These data suggested that POU4F1 regulated G1/S phase transition of cell cycle‐related pathway most profoundly in vitro. Consistently, in the TCGA database, BLBC patients with high POU4F1 expression showed enriched cell‐cycle related gene sets, the top of which was E2F target, followed by mitotic spindle and G2/M checkpoint (Figure 3F).

To investigate how POU4F1 regulated cell cycle distribution, we performed ChIP‐qPCR of POU4F1 and found that POU4F1 was enriched in the promoter of *CCND1* and *CDK2* (Figure 3G), suggesting that POU4F1 regulated cell cycle distribution through direct binding in the DNA sequence of *CCND1* and *CDK2*. These data suggested that POU4F1 drove the malignant progression of BLBC via activating cell‐cycle related pathways, mainly G1/S phase transition.

### 2.4 Targeting POU4F1 Activates Functional ERα Expression in BLBC

**Figure 4:**
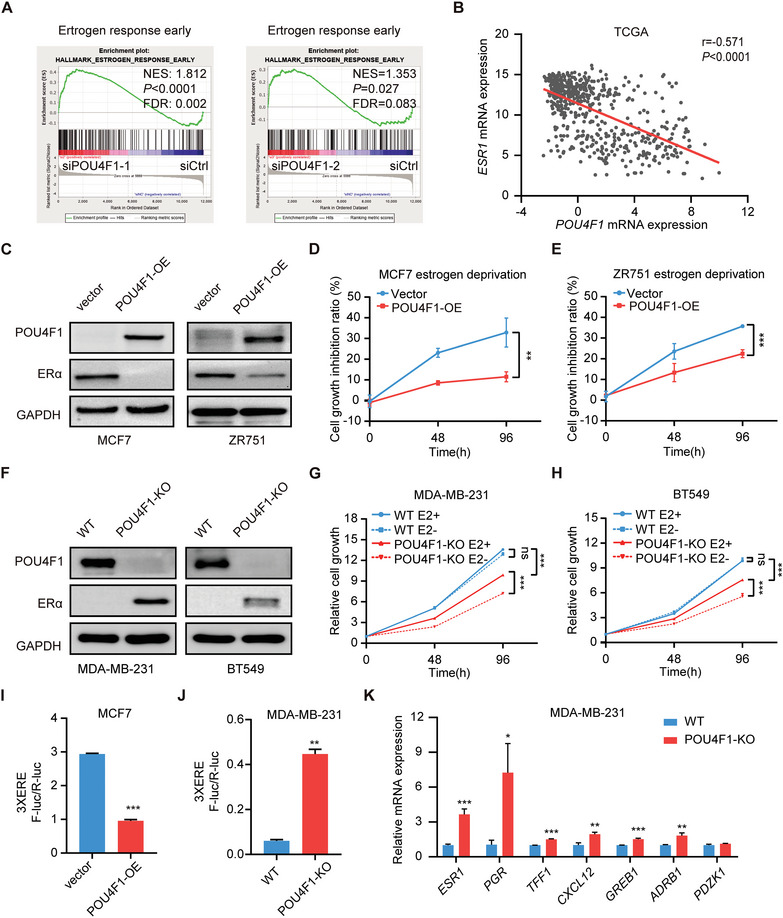
Targeting POU4F1 activates ERα expression and confers estrogen‐dependent growth of BLBC. A) GSEA of significant gene sets enriched in BT549 cells transfected with POU4F1 siRNAs. B) Scatter plot showing the Pearson correlation between *POU4F1* and *ESR1* mRNA expression of breast cancer patients in the TCGA cohort. Pearson's correlation coefficient r and two‐tailed *P* value were shown. C) Western blotting showing the protein expression of ERα in POU4F1‐overexpressed MCF7 and ZR751 cells. D,E) Cell growth inhibition ratio in POU4F1‐overexpressed MCF7 D) and ZR751 E) cells cultured in estrogen‐deprived medium. F) Western blotting showing the protein expression of ERα in POU4F1‐knockout MDA‐MB‐231 and BT549 cells. G,H) Cell growth curves of POU4F1‐knockout MDA‐MB‐231 H) and BT549 I) cells cultured in estrogen‐deprived medium. I,J) The luciferase reporter activities of 3xERE in POU4F1‐overexpressed MCF7 cells I) and POU4F1‐knockout MDA‐MB‐231 cells J). K) The mRNA expression of *ESR1* and its downstream genes in POU4F1‐knockout MDA‐MB‐231 cells, detected by qRT‐PCR. Data were presented as mean ± S.D., *n* = 3 D, E, G‐K). ns, no significance, **P < 0.01, ***P < 0.001 compared with vector D,E,I) or WT J) by two‐tailed Student's t‐test D, E, G‐K).

Emerging evidences have shown that cell subtype‐specific master regulators are indispensable for cell identity.^[^ ^7^, ^14^ ^]^ To determine whether the expression of POU4F1 plays a dominant role in BLBC cell identity, RNA‐sequencing data were further analyzed by gene set enrichment analysis (GSEA). The results revealed that estrogen early response was the significant gene set enriched in the BT549 cells with POU4F1 knockdown (**Figure** **4**A), indicating that estrogen response pathway was upregulated when knocking down POU4F1 in BLBC cells. Moreover, we observed a significant negative correlation between *POU4F1* expression and master transcription factors of ER positive breast cancer, such as *ESR1*, *FOXA1* and *GATA3*, in breast cancer patients from the TCGA and METABRIC cohorts (Figure 4B; Figure S6A,B, Supporting Information). To further investigate whether ectopic expression of POU4F1 may influence the phenotype of breast cancer, we stably expressed POU4F1 in the committed ER^+^ breast cancer cell lines, MCF7 and ZR751. We found that POU4F1 overexpression significantly decreased the mRNA and protein expression of ERα, and the luminal marker *CK18*, as well as increased the expression of the basal marker *CK14* (Figure 4C; Figure S6C,D, Supporting Information). Moreover, the inhibitory effect of estrogen deprivation on ER^+^ breast cancer cell growth was significantly reduced when POU4F1 was overexpressed (Figure 4D,E). These data suggested that overexpression of POU4F1 might reprogram the ER^+^ breast cancer cell into a BLBC‐like phenotype, as indicated by the diminished expression of ERα and acquired estrogen‐independent cell proliferation. On the contrast, we used the CRISPR/Cas9 system to knockout POU4F1 (POU4F1‐KO) in two BLBC cell lines, BT549 and MDA‐MB‐231. After POU4F1 depletion, the expression of ERα and *CK18* was upregulated in BT549 and MDA‐MB‐231, while *CK14* expression were decreased (Figure 4F,K; Figure S6E, Supporting Information). Consistently, silencing POU4F1 in BLBC cells not only decreased the cell proliferation in normal culture medium, but have a more obvious inhibitory effect in estrogen‐deprived culture medium, while wild‐type BLBC cell growth was estrogen‐independent, suggesting POU4F1 knockout results in the acquired estrogen‐dependent cell growth of BLBC cell lines (Figure 4G,H). More importantly, as ERα binds to estrogen response element (ERE) of target genes to regulate gene transcription and influence the cell commitment,^[^ ^15^ ^]^ we observed that the luciferase reporter activity of 3xERE was significantly decreased in POU4F1‐overexpressing MCF7 (Figure 4I). Moreover, the expression of downstream genes, including *PGR*, *TFF1*, *CXCL12*, *GREB1*, *PZDK1* and *ADRB1* was significantly reduced in POU4F1‐overexpressing MCF7 and ZR751 (Figure S6F,G, Supporting Information). On the contrary, the luciferase reporter activity of 3xERE was increased in POU4F1‐KO MDA‐MB‐231 cells, accompanied by the upregulation of the expression of ESR1 and its downstream genes (Figure 4J,K). Altogether, these data demonstrated that POU4F1 was required for BLBC cell identity and might contribute to the switch of lineage commitment in breast cancer cell lines.

### 2.5 POU4F1 Regulates ERα Expression through CDK2/EZH2 Axis

**Figure 5:**
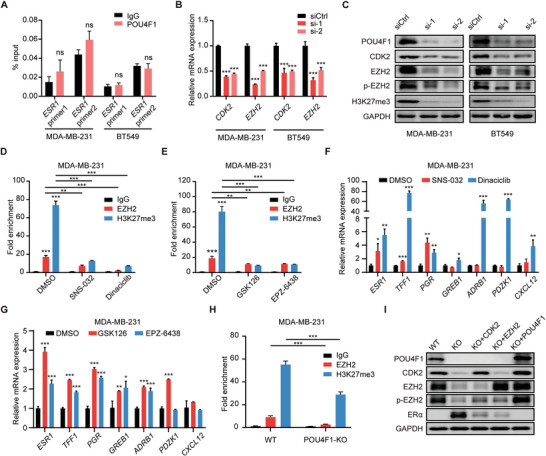
POU4F1 repressed ERα expression through CDK2/EZH2 axis. A) ChIP‐qPCR analysis of the enrichment of POU4F1 at the promoter of *ESR1* in MDA‐MB‐231 and BT549 cells. B,C) Indicated protein and mRNA expression in MDA‐MB‐231 and BT549 transfected with POU4F1 siRNAs and control siRNA. D, E) ChIP‐qPCR analysis of the enrichment of EZH2 and H3K27me3 at the promoter of *ESR1* in MDA‐MB‐231 treated with DMSO, 100 nM SNS‐032 and 25 nM Dinaciclib D) and 5 µM GSK126 and 10 µM EPZ‐6438 E) for 72 h. F,G) The mRNA expression of *ESR1* and its downstream genes in MDA‐MB‐231 cells treated with DMSO, 100 nM SNS‐032 and 25 nM Dinaciclib F) and 5 µM GSK126 and 10 µM EPZ‐6438 G) for 72 h. H) ChIP‐qPCR analysis of the enrichment of EZH2 and H3K27me3 at the promoter of ESR1 in POU4F1‐KO MDA‐MB‐231 cells. I) Western blotting showing the expression of indicated protein after rescue with CDK2, EZH2 and POU4F1 plasmid in POU4F1‐KO MDA‐MB‐231 cells. Data were presented as mean ± S.D., *n* = 3 A, B, D‐H). ns, no significance, *P < 0.05, **P < 0.01, ***P < 0.001 compared with IgG A),siCtrl B) and DMSO F,G) by two‐tailed Student's t‐test A) and two‐tailed one‐way ANOVA and Dunnett's B,F,G) or Bonferroni multiple‐comparisons test.

To investigate how POU4F1 regulates ERα expression, we first performed ChIP‐qPCR of POU4F1 in BLBC cell lines. However, we did not detect the binding of POU4F1 on the promoter of *ESR1* (**Figure** **5**A), suggesting that POU4F1 might not regulate ESR1 expression through direct DNA binding. In the RNA‐seq data, we surprisingly found that CDK2 and EZH2 were the differentially downregulated genes upon POU4F1 knockdown (Figure 3B), which was further validated by qRT‐PCR and western blotting (Figure 5B,C). Since previous study has demonstrated that ERα was a direct target of CDK2/EZH2 axis in triple‐negative breast cancer.^[^ ^16^ ^]^ CDK2‐mediated phosphorylation of EZH2 at Thr416 repressed ERα transcriptional activation by catalyzing trimethylation of histone H3 lysine 27 (H3K27me3).^[^ ^16^ ^]^ We treated MDA‐MB‐231 cells with CDK2 inhibitors, Dinaciclib and SNS‐032, as well as competitive EZH2 inhibitors,^[^ ^17^ ^]^ GSK126 and EPZ‐6438, respectively. CDK2 inhibitors did not influence EZH2 expression, but reduced the phosphorylation of EZH2 at Thr416 (Figure S6H, Supporting Information), while the EZH2 inhibitors reduced H3K27me3 expression (Figure S6I, Supporting Information). Both CDK2 inhibitors and EZH2 inhibitors can block the binding of EZH2 and H3K27me3 on *ESR1* promoter (Figure 5D,E; Figure S6J, Supporting Information), and thereby enhanced the expression of ERα and its downstream genes (Figure 5F,G; Figure S6K, Supporting Information). These data validated that CDK2‐mediated EZH2 phosphorylation catalyzed H3K27me3 modification in *ESR1* promoter and inhibited ESR1 expression in BLBC cell lines.

To further investigate whether POU4F1 regulates ERα expression through EZH2, we silenced POU4F1 in BLBC cells and observed that the phosphorylation level of EZH2 at Thr416 and the H3K27me3 expression were suppressed (Figure 5C). Furthermore, the binding of EZH2 and H3K27me3 on the promoter of ESR1 was decreased when POU4F1 was knockout (Figure 5H). However, when EZH2 or CDK2 expression was rescued by overexpressing EZH2 and CDK2 in POU4F1‐KO cells, the ERα expression was significantly reduced (Figure 5I). When POU4F1 was replenished in POU4F1‐KO cells, the expression of ERα was abolished (Figure 5I). These results suggested that POU4F1 regulated ERα expression through CDK2/EZH2 axis.

### 2.6 Targeting POU4F1 Confers the Anti‐estrogen Sensitivity of BLBC

**Figure 6:**
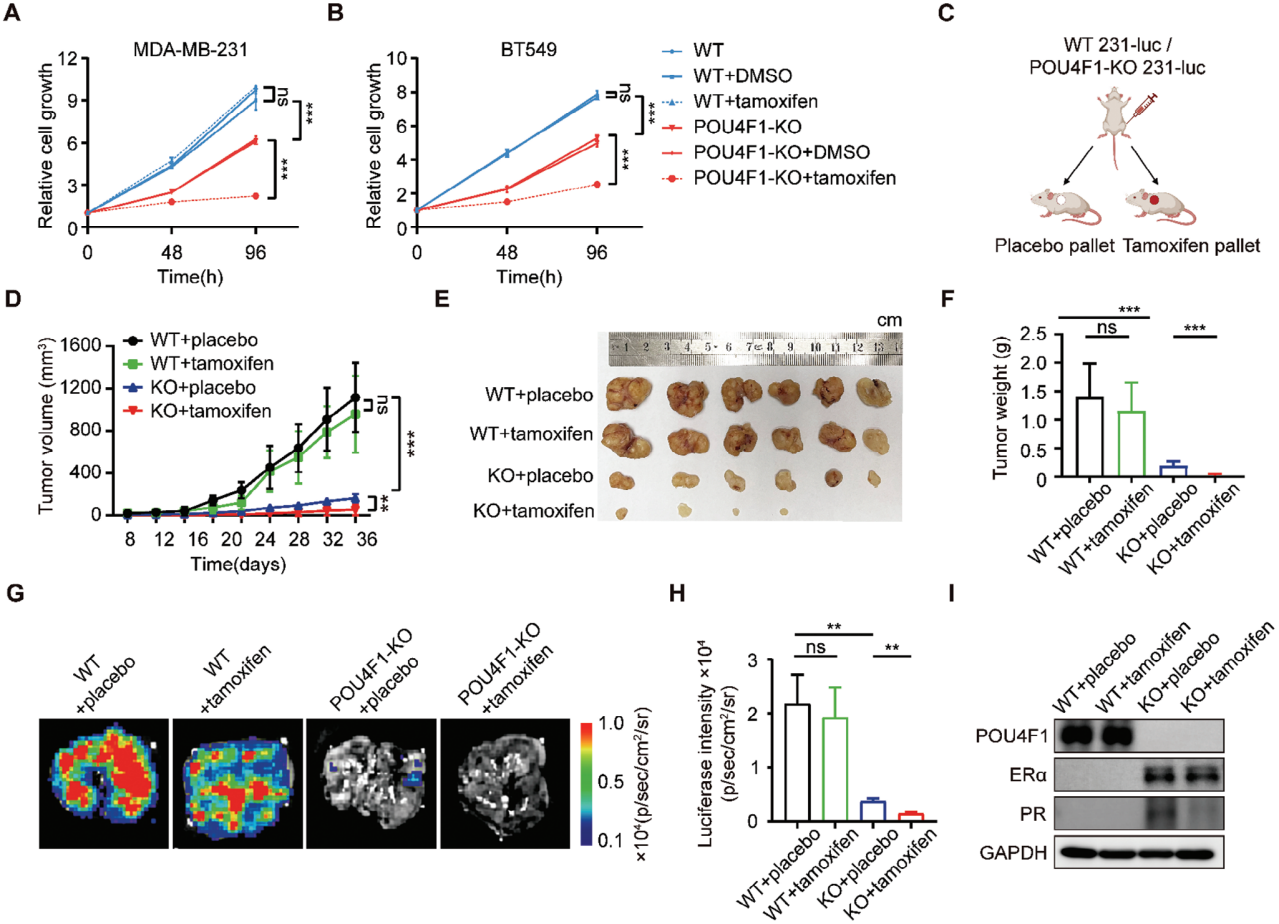
Targeting POU4F1 confers tamoxifen sensitivity of BLBC. A,B) Cell growth curves of WT and POU4F1‐knockout MDA‐MB‐231 A) and BT549 B) cells treated with 10 µM 4‐hydroxytamoxifen or DMSO. C) Schematic diagram of the mouse experiment. D–F) Tumor growth curves D), tumor images E) and tumor weight on day 40 F) of xenografts of WT and POU4F1‐KO MDA‐MB‐231 cells treated with subcutaneous embedding placebos or tamoxifen pallets. G,H) Representative images G) and quantitation analysis H) of bioluminescence signals of lung metastases isolated from tumor‐bearing mice treated with subcutaneous embedding placebos or tamoxifen pallets. I) Western blotting showing the indicated protein expression in xenograft of WT and POU4F1‐KO MDA‐MB‐231 cells treated with subcutaneous embedding placebos or tamoxifen pallets. Data were presented as mean ± S.D. A,B,D,F,H), *n* = 3 A,B) and *n* = 6 D–I). ns, no significance, **P < 0.01, ***P < 0.001 by two‐tailed Student's t‐test.

The anti‐tumor effect of endocrine therapy can only be achieved when the breast cancer is hormone receptor positive.^[^ ^18^ ^]^ To investigate whether POU4F1 knockout‐induced functional ERα re‐expression might resensitize BLBC cell lines to anti‐estrogen therapy, we treated the breast cancer cell lines with tamoxifen, an antagonist of ERα. POU4F1 overexpression rendered MCF‐7 cells less sensitive to tamoxifen (Figure S6L, Supporting Information), while POU4F1 depletion in MDA‐MB‐231 and BT549 cells inhibited cell growth under tamoxifen treatment, suggesting the acquisition of anti‐estrogen sensitivity (**Figure** **6**A,B). To further assess whether POU4F1 depletion in BLBC breast cancer cells confers tumor response to tamoxifen in vivo, we inoculated luciferase‐expressed wildtype MDA‐MB‐231 cells (WT) and POU4F1‐KO MDA‐MB‐231 into the mammary fatpads of female BALB/c nude mice, respectively, followed by subcutaneous embedding of tamoxifen or placebo pallets in mice (Figure 6C). We found that WT MDA‐MB‐231 xenografts did not respond to tamoxifen. However, knocking out POU4F1 in MDA‐MB‐231 xenografts not only inhibited tumor growth, but also enhanced the therapeutic response to tamoxifen treatment (Figure 6D–F). Furthermore, determined by bioluminescence signals in the lungs, lung metastasis was decreased in the mice bearing POU4F1‐KO xenografts, which were significantly reduced after tamoxifen treatment, while tamoxifen treatment did not inhibit lung metastasis in mice bearing WT xenografts (Figure 6G,H). Then the xenografts were collected to examine the expression of ERα and its downstream PR. We found that ERα and PR were re‐expressed in the POU4F1‐KO MDA‐MB‐231 xenografts, while PR was reduced after tamoxifen treatment (Figure 6I; Figure S6M, Supporting Information). Taken together, targeting POU4F1 not only inhibited the proliferation and metastasis of BLBC cells, but also rendered BLBC xenografts sensitive to endocrine therapy by re‐expression of ERα and reactivation of the downstream signaling.

### 2.7 Bivalent Chromatin in POU4F1 is Epigenetically Activated in BLBC

Genes can be transcriptionally activated by genetic or epigenetic alteration.^[^ ^19^ ^]^ To investigate the underlying mechanisms upon POU4F1 transcriptionally activation in BLBC, we first compared the gene alteration frequency across subtypes of breast cancer in the TCGA cohort. However, gene alteration frequency was low (1.75%) in BLBC and there was no difference between different subtypes (Figure S7A, Supporting Information). We then analyzed copy number values across subtypes of breast cancer patients in the TCGA cohort and breast cancer cell lines in the CCLE project. Similarly, the copy number values of *POU4F1* had no difference between subtypes of breast cancer patients and cell lines (Figure S7B,C, Supporting Information), suggesting that genetic alteration, including gain‐of‐function mutation and copy number amplification, did not contributed to POU4F1 expression in BLBC.

**Figure 7:**
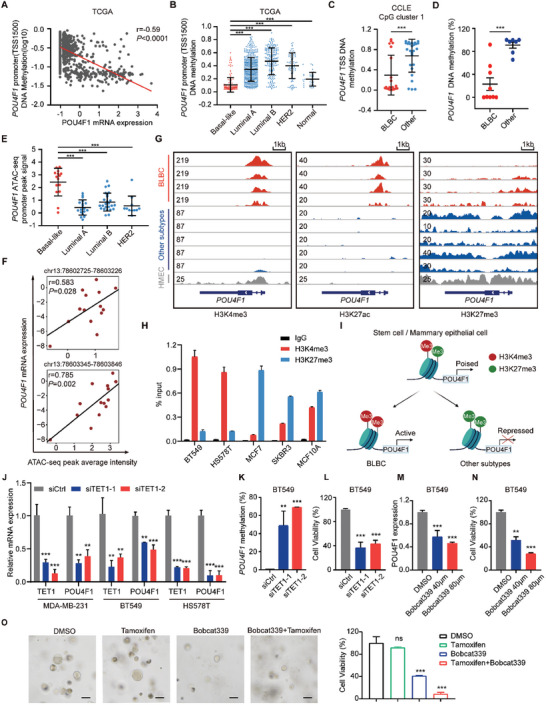
POU4F1 is epigenetically activated with bivalent chromatin re‐configuration and maintained by TET1 in BLBC. A) Scatter plot showing the Pearson correlation between *POU4F1* mRNA expression (Z‐scores) and its DNA methylation (log_10_) in breast cancer patients in the TCGA (*n* = 966) cohort. Pearson's correlation coefficient r and two‐tailed *P* value were shown. B) DNA methylation β values in *POU4F1* promoter across different subtypes of breast cancer patients using available WGBS data in the TCGA (Basal‐like: *n* = 168, HER2‐enriched: *n* = 78, Luminal A: *n* = 488, Luminal B: *n* = 197, Normal‐like: *n* = 35). C) DNA methylation β values of CpG clusters located in *POU4F1* transcription start site (TSS) in breast cancer cell lines from the CCLE project (BLBC: *n* = 21, other subtypes: *n* = 26). D) DNA methylation level of CpG island in *POU4F1* promotor in different breast cancer cell lines, detected by quantitative methylation‐specific PCR (qMSP). E) ATAC‐seq peak signals (log_2_((count+5)PM)‐qn) in *POU4F1* promoter across different subtypes of breast cancer patients in the TCGA (*n* = 69). F) Scatter plots showing the Pearson correlation between the ATAC‐Seq peak intensity of indicated gene locus of *POU4F1* and paired *POU4F1* mRNA expression of BLBC patients in the TCGA. Pearson's correlation coefficient r and two‐tailed *P* value were shown. G) ChIP‐seq peak signal of H3K4me3, H3K27ac and H3K27me3 across different breast cancer cell lines and normal mammary epithelial cells in *POU4F1* gene locus. The scale is shown in the upper left of each track. From top to bottom is BLBC (MDA‐MB‐468, MDA‐MB‐436, HCC1937, HCC1806), Luminal A (ZR751, MCF7), Luminal B (MDA‐MB‐361, UACC812), HER2^+^ (SKBR3) breast cancer cell lines and human mammary epithelial cells (HMEC). H) ChIP‐qPCR analysis of the enrichment of H3K4me3 and H3K27me3 at the *POU4F1* promoter. I) Schematic diagram showing bivalent chromatin re‐configuration of *POU4F1* in mammary epithelial cell, BLBC and other subtypes of breast cancer. J) POU4F1 expression in MDA‐MB‐231, BT549, HS578T cells transfected with TET1 siRNA or control siRNA, detected by qRT‐PCR. K,L) DNA methylation level of CpG island in *POU4F1* promotor K) and cell viability L) in BT549 transfected with TET1 siRNA, analyzed by qMSP and CCK8. M,N) POU4F1 expression M) and cell viability N) in BT549 treated with DMSO, Bobcat339 (40 µM) and Bobcat339 (80 µM) for 3 days. O) Representative images and quantification of cell viability of patient‐derived organoids of triple‐negative breast cancer treated with DMSO, 10 µM 4‐hydroxytamoxifen and 80 µM Bobcat339 for 9 days. Scale bar, 50 µm. Data were presented as mean ± S.D. *n* = 3 H,J–N), *n* = 5 O). ns, no significance, **P < 0.01, ***P < 0.001 compared with BLBC B–E), IgG H), siCtrl J–L) and DMSO M–O) by two‐tailed one‐way ANOVA and Dunnett's multiple‐comparisons test B,E, J–O) and by two‐tailed Student's t‐test C,D).

DNA methylation is the most common epigenetic alteration observed in tumors.^[^ ^20^ ^]^ We first investigated DNA methylation state in the promoter region of *POU4F1* in breast cancer patients and surprisingly found that high expression of *POU4F1* was significantly correlated with DNA hypomethylation in the promoter region of *POU4F1* in both TCGA and METABRIC cohorts (**Figure** **7**A; Figure S7D,E, Supporting Information). Moreover, the promoter of *POU4F1* exhibited significant hypomethylation in BLBC compared with other subtypes (Figure 7B; Figure S7F,G, Supporting Information). Consistently, the CpG clusters of *POU4F1* were significantly hypomethylated in BLBC cell lines, as determined by whole genome bisulfite sequencing in CCLE database and quantitative methylation‐specific PCR (Figure 7C,D; Figure S7H, Supporting Information). DNA hypomethylation was frequently accompanied by accessible chromatin, which is a hallmark of active promoter.^[^ ^21^ ^]^ Further analysis of the assay for transposase‐accessible chromatin using sequencing (ATAC‐Seq) of breast cancer patients showed that BLBC patients had higher chromatin accessibility in the *POU4F1* promoter and there was a significant positive correlation between the *POU4F1* expression and the intensities of the two ATAC‐seq peaks located in the transcription start site (TSS) region of *POU4F1* in BLBC patients (Figure 7E,F).

The functional state of chromatin has been defined largely by the covalent modifications of the histones, with their specific patterns serving as indicators of transcriptional activity.^[^ ^22^ ^]^ To further confirm that epigenetic activation of POU4F1 is responsible for its high expression in BLBC, we investigated the histone modification of *POU4F1* in different subtypes of breast cancer cell lines and normal breast epithelial cells. We unexpectedly found that the promoter of *POU4F1* maintained in a bivalent chromatin state in the normal breast epithelium cells, with concurrent presence of activated H3K4me3 and the repressive H3K27me3 chromatin modifications (Figure 7G), resembling the pattern observed in embryonic stem cells (ESCs) (Figure S8A, Supporting Information). Consistently, through analyzing the chromatin states in the TSS ± 1000 bp region of *POU4F1* across 57 cell types of human normal tissue profiled by the Epigenome Roadmap project,^[^ ^23^ ^]^ we found that over 70% cell types, such as ESC, epithelial tissue, breast epithelium etc., exhibit bivalent states, including bivalent TSS, flanking bivalent TSS, and bivalent enhancer, in the *POU4F1* TSS and its flanking regions (Figure S8B,C, Supporting Information). The chromatin configuration observed in the majority of normal cell types was consistent with their low mRNA expression of *POU4F1* (Figure S8C, Supporting Information). As previously reported,^[^ ^24^ ^]^ genes with bivalent chromatin states maintained at low expression level, but poised for quick transcriptional activation or silence and are prone to be DNA hypermethylation and transcriptional repression in cancer. In breast cancer cells, through analyzing the public available ChIP‐seq dataset,^[^ ^25^ ^]^ we found that the chromatin at the *POU4F1* promoter in BLBC cell lines was characterized by the increased binding of H3K4me3 and H3K27ac, representing *POU4F1* promoter was open for active transcription in BLBC (Figure 7G). However, other subtypes of breast cancer cell lines exhibited enhanced binding of H3K27me3 in *POU4F1* promoter, indicating the repressed transcription of *POU4F1* (Figure 7G), which was further validated by ChIP‐qPCR (Figure 7H). These findings suggest an epigenetic regulation model for cell‐fate switching, wherein POU4F1 is maintained in a bivalent chromatin state in normal breast epithelial cells and would undergo chromatin reconfiguration in different subtypes of breast cancer. Upon transformation and differentiation into BLBC, POU4F1 lose repressive H3K27me3 histone modification and became DNA hypomethylated, leading to transcriptional activation, while in other subtypes, POU4F1 lost H3K4me3 histone modification and became DNA hypermethylated, resulting in gene silencing (Figure 7I).

### 2.8 TET1 Maintains DNA Hypomethylation of POU4F1 and Contributes to Gene Activation in BLBC

DNA demethylation has emerged to play an important role in regulating the activation of bivalent promoters.^[^ ^24^, ^26^ ^]^ To further confirm that DNA hypomethylation was responsible for the high expression of POU4F1, we employed 5‐aza‐2′‐deoxycytidine (5‐Aza‐dC), an inhibitor of DNA methyltransferase, to treat normal MCF10A, luminal MCF7 and HER2^+^ SKBR3 cells. Upon 5‐Aza‐dC treatment, POU4F1 expression was significantly upregulated in these cells, suggesting that DNA hypomethylation contributed to POU4F1 expression (Figure S9A, Supporting Information). DNA hypomethylation is mediated by TET family, which oxidized 5‐methylcytosine (5mC) iteratively, followed by TDG excision.^[^ ^27^ ^]^ We first analyzed the correlation between TET1, TET2, TET3, TDG and POU4F1 in breast cancer patients in TCGA cohort. We found that TET1, TET3 and TDG showed positive correlation with POU4F1 (Figure S9B, Supporting Information). Then we compared their expression in different subtypes of breast cancer and found only TET1 was specifically highly expressed in BLBC (Figure S9C, Supporting Information). TET1 has been reported to mediate aberrant hypomethylation in TNBC/BLBC and promote tumor progression.^[^ ^28^ ^]^ We then used siRNA to silence TET1 in BT549, MDA‐MB‐231, and HS578T cells and found TET1 knockdown significantly enhanced the DNA methylation of *POU4F1*, leading to the downregulation of *POU4F1* (Figure 7J,K; Figure S9D, Supporting Information). In addition, the cell growth of BT549 and HS578T cells with TET1 knockdown was significantly inhibited, suggesting that TET1 contributed to POU4F1 DNA hypomethylation and its high expression in BLBC (Figure 7L; Figure S9E, Supporting Information). Moreover, upon TET1 knockdown, the expression of *ESR1* and its downstream genes were significantly upregulated (Figure S9F, Supporting Information). We further treated BLBC cell lines with Bobcat339, an inhibitor of TET1. Upon Bobcat339 treatment, POU4F1 was downregulated, and cell growth was inhibited, (Figure 7M,N; Figure S9G,H, Supporting Information). We then established patient‐derived organoid models of TNBC and found that Bobcat339 treatment significantly inhibited the organoid growth (Figure 7O). More importantly, the combination of TET1 inhibitor and tamoxifen significantly inhibited the growth of the patient‐derived organoid models of TNBC, compared with TET1 inhibitor or tamoxifen monotherapy (Figure 7O), suggesting that POU4F1‐targeted therapy combined with tamoxifen might be a potential therapeutic strategy for BLBC.

## 3 Discussion

In summary, we provided comprehensive evidence that POU4F1 was a BLBC‐specific TF that played a major role in regulating the biological and phenotypic characteristics of BLBC. POU4F1 was primarily documented as a TF contributing to the development and maintenance of sensory neurons,^[^ ^29^ ^]^ Transiently co‐expressing POU4F1 with NGN1 or NGN2 was sufficient to convert mouse and human fibroblasts into electrically active induced sensory neurons, suggesting the robust transcriptional regulation capability of POU4F1.^[^ ^30^ ^]^ Beyond its role in the nervous system, POU4F1, as an oncogene, has been attracted the attention in malignancies. POU4F1 promoted the survival of melanoma cells by activating the transcription of anti‐apoptotic genes such as Bcl‐2 and Bax, as well as induced drug resistance of melanoma cells through regulating the MEK/ERK pathway.^[^ ^31^ ^]^ In our study, we found that POU4F1 promoted the tumor growth and drove the malignant progression of BLBC, including proliferation, colony formation, migration and invasion, via activating cell‐cycle related pathways, which was consistent with the transcriptional signature hallmarks of BLBC as previously reported.^[^ ^2^, ^3^, ^32^ ^]^ These findings implicated that POU4F1 was a key TF of BLBC and may serve as a potential regulator of gene expression profiles in BLBC.

We found that POU4F1 directly bound in the promoter of *CCND1* and *CDK2* to regulated G1/S transition in BLBC. Further investigation found that CDK2/EZH2 axis was responsible for the POU4F1‐repressed ERα expression, thereby maintaining of cell identity of BLBC. In recent years, research have found that cell cycle closely regulates cell fate specification, which generally occur in G1 phage and associated with the CDK activity.^[^ ^33^ ^]^ Study has demonstrated that CDK2‐dependent phosphorylation of the histone methyltransferase MLL2 regulates the cell differentiation of pluripotent stem cells.^[^ ^34^ ^]^ In our study, POU4F1 upregulated the expression of CDK2 in BLBC, followed by the phosphorylation of EZH2 and the modification of H3K27me3 on the *ESR1* promoter, leading to the suppressed expression of ERα. When CDK2 and EZH2 was recovered in POU4F1‐KO BLBC cells, ERα was re‐expressed and the downstream signaling was re‐activated, resulting in the conversion between basal‐like and luminal‐like phenotype, suggesting the regulatory role of cell cycle in tumor cell identity.

Long‐standing lack of effective therapeutic strategies other than chemotherapy has contributed to BLBC being the subtype with the poorest clinical outcomes,^[^ ^35^ ^]^ which warrant the investigation of new therapeutic targets. TNBC has different classification standard with BLBC, however, the research findings on BLBC might offer new insights into TNBC due to the high overlap between these two subtypes. Previous studies have been reported that histone deacetylase inhibitors or EZH2 inhibitors could result in a phenotypic switch from BLBC into ER^+^ breast cancer, leading to the acquisition of sensitivity to hormone therapy,^[^ ^16^, ^36^ ^]^ but the master regulator remained unclear. Importantly, our findings demonstrated that depletion of POU4F1 by CRISPR/Cas9 system or inhibition its downstream signaling by CDK2 inhibitors and EZH2 inhibitors could induce functional ERα expression in the committed BLBC cell lines, which were also belong to TNBC subtype, rendering them targetable by tamoxifen. These findings uncovered the master TF regulating the cell identity, supporting that converting BLBC into ERα‐positive breast cancers by targeting POU4F1 is a therapeutically actionable approach for BLBC patients, even TNBC patients. However, since BLBC and TNBC are not entirely interchangeable, further investigations might be necessary to validate the applicability.

A wide body of evidence has indicated that epigenetic alternation involves in oncogenesis by activating or silencing of oncogenes or tumor suppressor genes.^[^ ^19a^ ^]^ The bivalent chromatin modification state is thought to poise important regulatory genes for expression or repression during cell‐lineage specification, based on the observation that bivalent chromatin in ESCs is highly enriched in developmental TFs that are activated and functional only during differentiation.^[^ ^24^, ^37^ ^]^ Being initially discovered in ESCs, increasing evidences established that bivalent genes play critical roles in malignant transformation, including hematopoietic malignancy and solid tumors.^[^ ^7^, ^38^ ^]^ The bivalent gene can undergo transcription activation or repression, accompanied by de novo methylation of DNA, depending on different cancer types.^[^ ^39^ ^]^ In this study, we demonstrated that the promoter of POU4F1 was in a bivalent chromatin state. When the mammary epithelial cells were transformed into BLBC, bivalent POU4F1 lost H3K27me3, resulting in gene activation, while in other breast cancer subtypes, bivalent POU4F1 lost H3K4me3, resulting in gene silence. The regulatory mechanisms underlying the activation of bivalent chromatin are not yet fully understood, with DNA demethylase and histone methyltransferases playing a certain role in this process.^[^ ^24a^ ^]^ We found that DNA demethylase TET1 maintained the DNA hypomethylation of POU4F1 and contributed to its high expression. Treating organoid with TET1 inhibitor inhibit cell growth and showed synergistic effect with tamoxifen. Although targeting TFs poses challenges, this discovery provides a compelling theoretical foundation for exploring the potential of utilizing epigenetic drugs as well as its combination with endocrine therapy in the treatment of BLBC.

In conclusion, we identified POU4F1 as a BLBC‐specific hyperactive TF, the expression of which is associated with tumor growth and metastasis, leading to poor clinical outcomes. Targeting POU4F1 inhibited malignant phenotypes as well as reactivated ERα expression in BLBC, conferring anti‐estrogen sensitivity. Thus, targeting POU4F1 might be explored as a potential therapeutic strategy combined with endocrine therapy for the treatment of BLBC in the future.

## 4 Experimental Section

### Patient Samples

Primary breast tumor samples for immunohistochemistry and organoids were obtained from patients at Sun Yat‐Sen Memorial Hospital, Sun Yat‐Sen University (Guangzhou, China) following surgical resection from 2016 to 2024. Breast cancer subtype was verified by immunohistochemistry by pathologists. All samples were collected from patients with informed consent, and all related procedures were performed with the approval of the internal review and ethics boards of the Sun Yat‐Sen Memorial Hospital.

### Cell Lines

Human breast cancer cell lines (MDA‐MB‐231, BT549, HCC1937, HS578T, MDA‐MB‐468, MDA‐MB‐453, MDA‐MB‐436, SUM149PT, SUM159PT, T47D, ZR7530, MCF‐7, ZR751, BT474 and SKBR3), human breast epithelial cell line (HMLE and MCF‐10A) and human embryonic kidney cell line 293T were purchased from American Type Culture Collection. All cell lines were grown according to standard protocols, authenticated by short tandem repeat profiling and were tested negative for mycoplasma contamination.

### Public Data Acquisition

Analyzed public datasets of breast cancer patient samples and cell lines are as following: the TCGA cohort, METABRIC cohort,^[^ ^40^ ^]^ CCLE project,^[^ ^41^ ^]^ Heiser dataset,^[^ ^13a^ ^]^ Neve dataset,^[^ ^13b^ ^]^ Epigenome Roadmap project^[^ ^23^ ^]^ and ChIP‐seq of breast cancer cell lines (GSE85158).^[^ ^25^ ^]^ Data were downloaded through the NCI's Genomic Data Commons (GDC), GEO, UCSC Xena,^[^ ^42^ ^]^ cBio Cancer Genomics Portal^[^ ^43^ ^]^ and Broadinstitute portal.

### ELMER Analysis

The ELMER analysis was performed as previously described.^[^ ^12b^ ^]^ Briefly, DNA methylation, RNA‐seq and clinical data of breast cancer patients in the TCGA were downloaded from the GDC (https://portal.gdc.cancer.gov/) using R package TCGAbiolinks and unsupervised analysis model was performed. First, probes located at least 2 kb away from any TSS were selected and distal probes with significant differential DNA methylation were identified. Then putative target genes for differentially methylated distal probes and enriched motifs were identified, follow by the identification of master regulator TF of breast cancer subtypes.

### Cell Growth Assay

Cancer cells in complete cell culture medium, hormone‐deprived medium, or culture medium supplemented with DMSO, 4‐hydroxytamoxifen or Bobcat339 were seeded in 96‐well plates at 1000–2000 cells well^−1^ and measured by Cell Counting Kit‐8 (CCK8, Cat# CK04, Dojindo Laboratories) according to the manufacturer's instructions in day 0, 2, 4 or 72 h. Relative cell growth was determined by normalizing to cell numbers at day 0.

### EdU Incorporation Assay

The 5‐Ethynyl‐2‐deoxyuridine (EdU) incorporation assay was conducted using BeyoClick EdU Cell Proliferation Kit with Alexa Fluor 488 (Cat# C0071, Beyotime) according to the manufacturer's instructions. Briefly, cells were seeded into a 96‐well plate and incubated with diluted EdU reagent (final concentration 10 µM) at 37 °C for 2 h. After fixed with 4% paraformaldehyde (PFA) and permeated with 0.3% Triton X‐100, cells were incubated with click reaction liquid at room temperature (RT) for 30 min. DAPI was applied to counterstain the nuclei for 15 min and the cells were observed and photographed under a fluorescence microscope.

### Migration and Invasion Assays

Boyden chambers were used to assess the migration and invasion of breast cancer cells (8 µm pore size, Cat# 353 097, Corning). For invasion assay, Matrigel (Cat# 356 234, Corning) diluted with serum‐free DMEM medium at a 1:10 ratio was precoated in the chambers and solidified for 1 h at 37 °C to form a matrix barrier. 1 × 10^5^ tumor cells were plated on the upper chamber with serum‐free medium, and medium containing 10% FBS was used in the lower chamber. The migration and invasion time was controlled to within 12 h to avoid the effect of proliferation. In POU4F1 knockdown experiment, the migration time of MDA‐MB‐231, BT549, HS578T, HCC1806 MB468 and HCC1937 were 3, 4, 6, 8, 6, and 8 h respectively and the invasion time of MDA‐MB‐231, BT549, HS578T were 6, 12, and 12 h respectively. In POU4F1 overexpression experiment, the migration and invasion time of MDA‐MB‐231, BT549, HS578T were 3 and 6 h respectively. After incubation at 37 °C, migrated cells and invaded cells were fixed, stained with 0.1% crystal violet, and photographed under a microscope. Five different fields of each well were counted at a magnification of 200×.

### Colony Formation Assay

For colony formation assay, 1000–2000 cells were seeded in 6‐well plates and cultured for 10–14 days. After fixed and staining with 0.1% crystal violet, the number of colonies was counted by vSpot Spectrum (AID). For adherent‐independent colony formation assay, 0.6% base agarose and 0.35% top agarose were prepared by diluting corresponding concentration of 2× agarose with 2× complete medium at 1:1 ratio. After 1 mL of the base agarose as solidified in 12 well plate, 1000–5000 cells well^−1^ of cell suspension mixed with top agarose was plated and incubated at 37 °C humidified incubator for 3–4 weeks.

### Cell Cycle Analysis

After siRNA or plasmid transfection for 48 h, cells were harvested and fixed with 70% ethanol at 4 °C overnight. Cells were then treated with RNase A (100 µg mL^−1^) at 37 °C for 5 min. DNA of the cells was stained with Propidium Iodide solution (final concentration being 25 µg mL^−1^). Samples were then analyzed using flow cytometer (BD Biosciences) and all data were processed by ModFit.

### Cell Apoptosis Analysis

After siRNA transfection for 36 h, cells were harvested, wash and resuspended in binding buffer. Apoptotic cells were assessed by Annexin V‐FITC/PI staining (Cat# 640 914, BioLegend) according to the manufacturer's instructions, followed by flow cytometry analysis (Beckman).

### Immunohistochemistry and Hematoxylin‐Eosin (H&E) Staining

The paraffin‐embedded samples were sectioned at 4‐µm thickness. Antigen retrieval was performed using a pressure cooker for 5–10 min in EDTA buffer (pH 9.0) to remove the aldehyde links formed during the initial fixation of tissues. Endogenous peroxidase was eliminated by 3% hydrogen peroxide. The non‐specific binding was blocked with 5% BSA for 30 min at RT, then the tissues were incubated with antibodies against POU4F1 (Cat# ab245230, abcam, 1:200), ERα (Cat# ab241577, abcam, 1:500) and Ki67 (Cat# ZM‐0166, ZSGB‐BIO, ready to use) overnight at 4 °C. The slices were then washed and incubated with HRP‐conjugated secondary antibodies (Cat# PV‐6000, ZSGB‐BIO, ready to use). After rigorous washing by PBS, immunodetection was performed using DAB (DAKO) according to the manufacturer's instructions and nuclei was counterstained with hematoxylin. The stained slides were imaged under an optical microscope (NIKON) with at least five different fields and measured by immunoreactive scores (IRS) based on the staining intensity and area.^[^ ^44^ ^]^ The staining intensity (0 = no staining, 1 = weak staining, 2 = moderate staining, 3 = strong staining) and the percentage of stained tumor cells (0 = 0%, 1 = 1%–24%, 2 = 25%–49%, 3 = 50%–74%, and 4 = 75%–100%) were multiplied, resulting in a final IRS. For H&E staining, the sections were immersed in hematoxylin solution for 5 min, rinsed with tap water for 30 min and then counterstained with eosin solution for 1–2 min.

### Inhibitor Treatment

The small‐molecule compounds were purchased from Selleck (SNS‐032, Dinaciclib, GSK126, EPZ‐6438 (Tazemetostat), Bobcat339) and Sigma–Aldrich (5‐aza‐2′‐deoxycytidine). For cell treatment, cells were seeded in 6 well plate at 30%–50% confluency overnight and treated with SNS‐032 (100 nM), Dinaciclib (25 nM), GSK126 (5 µM), EPZ‐6438 (10 µM) and 5‐Aza‐dC (10 µM) for 3 days, with Bobcat339 (40 and 80 µM) for 4 days. Control cells were treated with the corresponding amount of vehicle (DMSO).

### siRNA Transfection, shRNA Transduction and Plasmid Overexpression

Control siRNA and siRNAs targeting POU4F1 or TET1 were purchased from RiboBio. For siRNA transfection, 1 × 10^5^ cells were plated in 6‐well plates overnight and transfected with specific siRNA duplexes with Lipofectamine 3000 (Cat# L3000015, Thermo Fisher Scientific) or Lipofectamine RNAiMAX (Cat# 13 778 150, Thermo Fisher Scientific) according to the manufacturer's instructions. For shRNA‐mediated gene silencing and plasmid‐mediated gene overexpression, sequences were cloned into lentiviral vector plasmid pLKO.1 (Cat# 10 878, Addgene) and pCDH‐CMV‐EF1‐copGFP‐T2A‐Puro (IGEBio) respectively. Viral particles were produced by co‐transfected target plasmid with psPAX2 and pMD2G vectors (Cat# 12 259 and Cat# 12 260, Addgene) into HEK293T cells. Lentiviral supernatant was collected at 48 h post‐transfection, filtered through 0.45 µm filters and were used to transduce cancer cells seeded in 6‐well plates overnight at 37 °C with 5 mg mL^−1^ polybrene. 48 h post‐infection, cells were selected using puromycin (2 µg mL^−1^) for 2 weeks. The oligonucleotide sequences of siRNAs and shRNAs were listed in Table S4 (Supporting Information).

### Gene Knockout using CRISPR/Cas9 System

sgRNA sequences targeting POU4F1 were designed using Broad Institute GPP sgRNA Designer (https://portals.broadinstitute.org/gpp/public/analysis‐tools/sgrna‐design). sgRNA and complementary oligos were synthesized, annealed, phosphorylated and cloned into lentiCRISPRv2 (Cat# 52 961, Addgene),^[^ ^45^ ^]^ followed by transformed into Stbl3. Cloning was confirmed via Sanger sequencing. Lentiviral particles were generated, and cells were transduced and selected as mentioned above. Individual clones were allowed to growth and verified by immunoblotting. The oligonucleotide sequences of sgRNA were listed in Table S4 (Supporting Information).

### RNA Extraction and qRT‐PCR Analysis

Total RNA was extracted from cell lines by using Trizol reagent and complementary DNA was generated by the PrimeScript RT Master Mix (Cat# RR036A, Takara). Quantitative real‐time PCR (qRT‐PCR) was performed on a LightCycler 480 instrument (Roche) using TB Green Premix Ex Taq II (Cat# RR820A, Takara) according to the manufacturer's instruction. Gene expression was normalized to GAPDH or ACTB. The primer sequences were listed in Table S5 (Supporting Information).

### Quantitative Methylation‐Specific PCR (qMSP)

Total DNA was extracted using the DNA extraction kit (Cat# D0063, Beyotime). 500 ng DNA was bisulfite converted using EZ DNA Methylation‐Gold Kit (Cat# D5005, Zymo Research) according to the manufacturer's protocol. Two sets of qMSP primers were designed using MethPrimer^[^ ^46^ ^]^ (http://www.urogene.org/methprimer): one for unmethylated and the other for methylated DNA sequences (Table S6, Supporting Information). PCR reaction was performed on a LightCycler 480 instrument (Roche) using SYBR Green master mix (Cat# Q712, Vazyme) according to the manufacturer's instruction. The DNA methylation level of CpG island was calculated using the following equation: Methylated DNA(%) = 100 / [1 + 2^ΔCt^], where ΔCt = mean Ct methylated – mean Ct unmethylated.^[^ ^47^ ^]^

### RNA Sequencing and Data Analysis

Library construction and sequencing was performed at KangChen Bio‐tech (Shanghai, China). High‐throughput sequencing was performed using NovaSeq 6000 (Illumina, Inc.). Sequencing raw reads were preprocessed by filtering out ribosomal RNA reads, sequencing adapters, short‐fragment reads and other low‐quality reads. Hisat2 (v.2.0.4) was used to map the cleaned reads to the human GRCh37 reference genome with two mismatches. After genome mapping, StringTie (v.1.3.0) was run with a reference annotation to generate fragments per kilobase of transcript per million mapped reads (FPKM) for known gene models. Genes with *P* value < 0.05 and fold change > 1.5 were identified as differentially expressed genes. The sequencing data were deposited in the National Center for Biotechnology Information (NCBI) Gene Expression Omnibus (GEO) public database with accession number GSE261462.

### Western Blotting

Protein was extracted from the cells with RIPA buffer supplemented with protease and phosphatase inhibitor cocktail (Cat# 78 440, Thermo Fisher Scientific). The protein concentration was measured by Pierce BCA Protein Assay kit (Cat# 23 225, Thermo Fisher Scientific). Quantified protein lysates were resolved on SDS–PAGE gels, then transferred to polyvinylidene difluoride membranes. The primary antibodies against POU4F1 (Cat# ab81213 or Cat# ab245230, abcam), ERα (Cat# 8644, Cell Signaling Technology), Phospho‐Rb (Cat# 9308, Cell Signaling Technology), E2F2 (Cat# ab138515, abcam), CCND1 (Cat# 2978, Cell Signaling Technology), CDK2 (Cat# ab32147, abcam), Phospho‐CDK2 (Thr160)(Cat# 2561, Cell Signaling Technology), EZH2 (Cat# 5246, Cell Signaling Technology), PR (Cat# 8757, Cell Signaling Technology), H3K27me3 (Cat# 9733, Cell Signaling Technology), Phospho‐EZH2 (Thr416)(AF3585, Affinity Biosciences), GAPDH (Cat# 5174, Cell Signaling Technology) were used. HRP‐conjugated secondary anti‐rabbit or mouse antibody (Cat# 7074 or Cat# 7076, Cell Signaling Technology) was used and the antigen–antibody reaction was visualized using an enhanced chemiluminescence assay (Cat# 34 577, Thermo Fisher Scientific).

### Luciferase Reporter Assay

3×ERE was cloned into firefly luciferase reporter vector pGL3‐Promoter (Promega). The ERE sequence was as follow: GGTCACAGTGACCTGCGGATCCGCA. Luciferase reporter activity was measured by the Dual‐Luciferase Reporter Assay System (Cat# E1910, Promega) using microplate reader (TECAN). Firefly luciferase activity was normalized to Renilla luciferase activity for each sample.

### ChIP‐qPCR

ChIP‐qPCR was performed as previously described.^[^ ^48^ ^]^ Briefly, cells were washed with PBS, crosslinked with 1% formaldehyde for 10 min at RT and neutralized by glycine (1.25 M), followed by lysed and sonicated in Bioruptor Sonicator (Diagenode, USA). The resulting whole cell extract was incubated with H3K27me3 (Cat# 9733, Cell Signaling Technology), H3K4me3 (Cat# ab8580, abcam), EZH2 (Cat# 5246, Cell Signaling Technology), POU4F1 (Cat# ab245230, abcam) and normal rabbit IgG (Cat# 2729, Cell Signaling Technology) overnight at 4 °C and then Protein G magnetic beads (Invitrogen) was added for additional 4 h. The bound complexes were eluted from the beads and inverse‐crosslinked at 65 °C overnight. The enriched DNA was purified by treatment with Rnase A, proteinase K and HiPure DNA Clean Up Kit (Cat# D2141, Magen Biotechnology) and then subjected to qPCR analysis. The primers were listed in Table S6 (Supporting Information).

### Patient‐Derived Tumor Organoids

TNBC organoids were derived from fresh surgical specimens of TNBC patients and cultured based on previously described methods.^[^ ^49^ ^]^ Fresh breast cancer tissues were cut into small pieces and were digested in collagenase A solution with ROCK inhibitor on a shaker at 37 °C for 1 h. The digested tissue suspension was passed through a 100 µm filter and washed at least three times with PBS. Cell pellets were resuspended in Basement Membrane Extract (BME) type‐2 (Cat# 3533‐005‐02, R&D Systems) and solidified on prewarmed 24‐well culture plates at 37 °C for 20 min. The organoids were cultured in breast cancer organoid medium and maintained at 37 °C in a 5% CO2‐humidified atmosphere.^[^ ^49^ ^]^ For drug experiments, organoids were digested into single cells with TrypLE (Gibco) at 37 °C for 20 min before plating into wells of 96‐well plate (5000 cells in 5 µl BME /well). Drugs were added 48 h after plating in a final concentration of Bobcat339 (80 µM) and 4‐hydroxytamoxifen (10 µM). Treatments were renewed every three days. Cells were grown in the presence of drugs or DMSO for 9 days, and viability of organoids were measured by CCK8.

### Animal Xenograft Experiment

Four‐week‐old BALB/c‐nude mice were obtained from the Laboratory Animal Center of the Sun Yat‐Sen University (Guangzhou, China) and housed under standard conditions at the specific‐pathogen‐free (SPF) animal care facility of the Center of Experimental Animals of Cancer Center, Sun Yat‐sen University. Mice were randomly assigned to each group for different treatments. 2.5 × 10^6^ MDA‐MB‐231 or 1 × 10^6^ MDA‐MB‐231‐luc cells were orthotopically injected directly into the fourth pair mammary fat pads of mice (n = 6 per group) in 0.1 mL of sterile PBS. For therapeutic studies, tamoxifen pellets (5 mg pellet^−1^, Cat# SE‐361, Innovative Research of America) or placebo pellets (5 mg pellet^−1^, Cat# SC‐111, Innovative Research of America) were subcutaneously embedded. The tumors were measured every 4 days, and tumor volumes were calculated using the following formula: volume = length × width^2^ × 0.5. When the experiments were terminated, all mice were sacrificed, and tumor tissues and lung tissue were dissected for analyses. Lung‐metastasis was examined by bioluminescence or histological analysis. All experimental procedures were approved by the Institutional Animal Care and Use Committee of Sun Yat‐Sen University. The study was compliant with all relevant ethical Regulations regarding animal research.

### Statistical Analysis

IBM SPSS Statistics 25 and GraphPad Prism 8.0 were used to analyze and present data. Details of the statistical analysis for each experiment can be found in the relevant figure legends, text or method. Independent sample Student's t‐tests were used to calculate the *P* values between two groups. One‐way ANOVA and Dunnett's or Bonferroni multiple‐comparisons test were performed to calculate the statistical differences among more than two groups. Correlation analysis was performed using Pearson correlation coefficient. Kaplan‐Meier survival curves were plotted, and *P* values were calculated using univariate cox regression analysis or log‐rank test. X‐tile statistical software was used to define an optimal cut‐off value to group clinical samples. Chi‐square Test was used to assess the correlations between POU4F1 expression and clinical parameters. Multivariable Cox regression analysis was used to determine the independent prognostic factors. All experiments were performed at least three times. For all tests, *P* values were two‐sided. *P* values less than 0.05 were considered statistically significant.

## Conflict of Interest

The authors declare no conflict of interest.

## Author Contributions

J.Z., N.M., and L.L., contributed equally to this work. D.H., Y.N., and L.Y., designed and supervised the study. J.Z., N.M., L.L., W.D., J.W., X.Z., L.T., Y.H., H.L., W.Z., X.Y., X.Z., W.Z., C.X. and J.Z. performed experiments and acquired data. J.Z., N.M. and L.L analyzed data. E.S., Y.N., and D.H. provided patient samples for clinical data analysis. L.L and W.D. established the patient‐derived organoid models. J.Z., N.M., L.Y., Y.N., and D.H. wrote the paper. All authors read and approved the final manuscript.

## Supporting information

Supporting Information

Supporting Information

## Data Availability

The data that support the findings of this study are available in the supplementary material of this article.
