# Supplementary material for: Activation of Bivalent Gene POU4F1 Promotes and Maintains Basal‐like Breast Cancer
Source: Adv Sci (Weinh). 2024 Mar 16;11(20):2307660. doi: 10.1002/advs.202307660 (PMC11132042; doi:10.1002/advs.202307660)
Supplement: Supplementary file 1 — Supporting Information [file ADVS-11-2307660-s002.pdf]

Supporting Information

for *Adv. Sci.*, DOI 10.1002/advs.202307660

Activation of Bivalent Gene POU4F1 Promotes and Maintains Basal-like Breast Cancer

Jiahui Zhang, Nanyan Miao, Liyan Lao, Wen Deng, Jiawen Wang, Xiaofeng Zhu, Yongsheng Huang, Huayue Lin, Wenfeng Zeng, Wei Zhang, Luyuan Tan, Xiaoqing Yuan, Xin Zeng, Jingkun Zhu, Xueman Chen, Erwei Song, Linbin Yang, Yan Nie* and Di Huang**

Activation of Bivalent Gene POU4F1 Promotes and Maintains Basal-like Breast Cancer

Jiahui Zhang[#], Nanyan Miao[#], Liyan Lao[#], Wen Deng, Jiawen Wang, Xiaofeng Zhu, Yongsheng Huang, Huayue Lin, Wenfeng Zeng, Wei Zhang, Luyuan Tan, Xiaoqing Yuan, Xin Zeng, Jingkun Zhu, Xueman Chen, Erwei Song, Linbin Yang, Yan Nie* and Di Huang**

J.Zh., N.M., L.L., J.W., X.Zh., Y.H., H.L., W.Z., W.Z., L.T., X.Y., X.Z., J.Zh, C.X., E.S., L.Y., Y.N., D.H.

Guangdong Provincial Key Laboratory of Malignant Tumor Epigenetics and Gene Regulation, Guangdong-Hong Kong Joint Laboratory for RNA Medicine, Sun Yat-sen Memorial Hospital, Sun Yat-sen University

Breast Tumor Center, Sun Yat-sen Memorial Hospital, Sun Yat-sen University Guangzhou, 510120, China

E-mail: yanglb8@mail.sysu.edu.cn, nieyan7@mail.sysu.edu.cn, huangd63@mail.sysu.edu.cn

N.M.

Department of Plastic Surgery, Sun Yat-Sen Memorial Hospital, Sun Yat-Sen University. Guangzhou, 510120, China

W.D.

Center for Biotherapy, Sun Yat-sen Memorial Hospital, Sun Yat-sen University Guangzhou, 510120, China

Y.H.

Cellular & Molecular Diagnostics Center, Sun Yat-Sen Memorial Hospital, Sun Yat-Sen University

Guangzhou, 510120, China

[#]These authors contributed equally.

Keywords: POU4F1, basal-like breast cancer, bivalent chromatins, transcription factors, endocrine therapy

Supplementary Figures

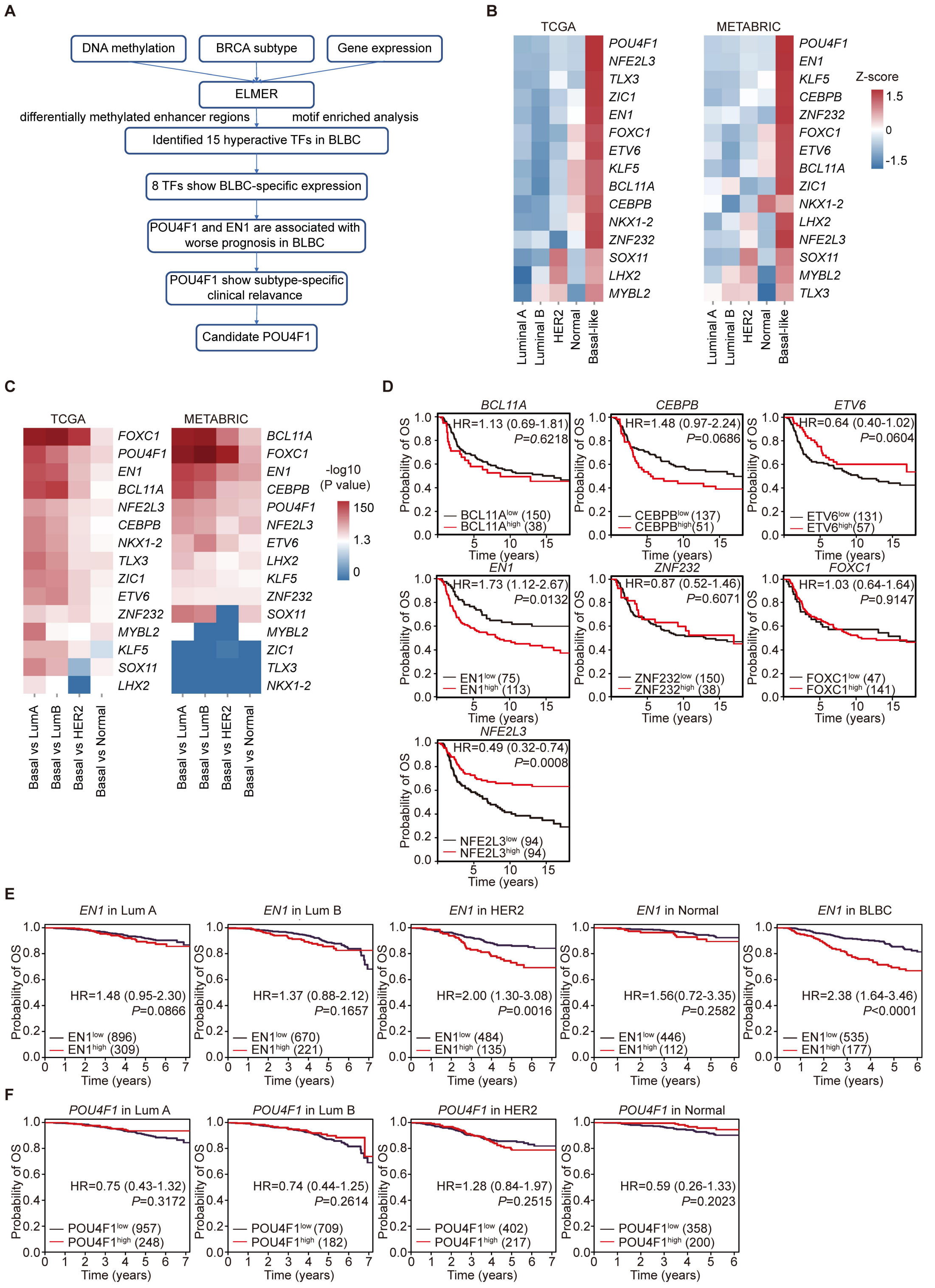

Figure S1. Screening and identification of POU4F1 as a hyperactive TF crucial for BLBC. A) Flowchart of identifying and screening of the crucial TFs of BLBC among

15 candidate hyperactive TFs. B) Heatmaps showing mRNA expression of 15 candidate TFs across PAM50 subtypes of breast cancer patients in the TCGA (Basal-like: n = 171, HER2-enriched: n = 78, Luminal A: n = 499, Luminal B: n = 197, Normal-like: n = 36) and METABRIC (Basal-like: n = 199, HER2-enriched: n = 220, Luminal A: n = 679, Luminal B: n = 461, Normal-like: n = 140) cohorts. C) Heatmaps showing statistical P values of *POU4F1* mRNA expression between indicated comparison groups of breast cancer patients in the TCGA and METABRIC cohorts. D) Kaplan-Meier survival plots showing overall survival (OS) of BLBC patients in the METABRIC cohort with high or low expression of candidate TFs. Hazard ratio (HR) and 95% confidence interval (95% CI) were shown. E, F) Kaplan-Meier survival plots showing OS in different subtypes of breast cancer patients with high or low *EN1* (E) or *POU4F1* (F) mRNA expression in the TCGA and SCAN-B cohorts. Hazard ratio (HR) and 95% confidence interval (95% CI) were shown. P value was determined by two-tailed one-way ANOVA and Bonferroni multiple-comparisons test (C) and univariate cox regression analysis (D-F).

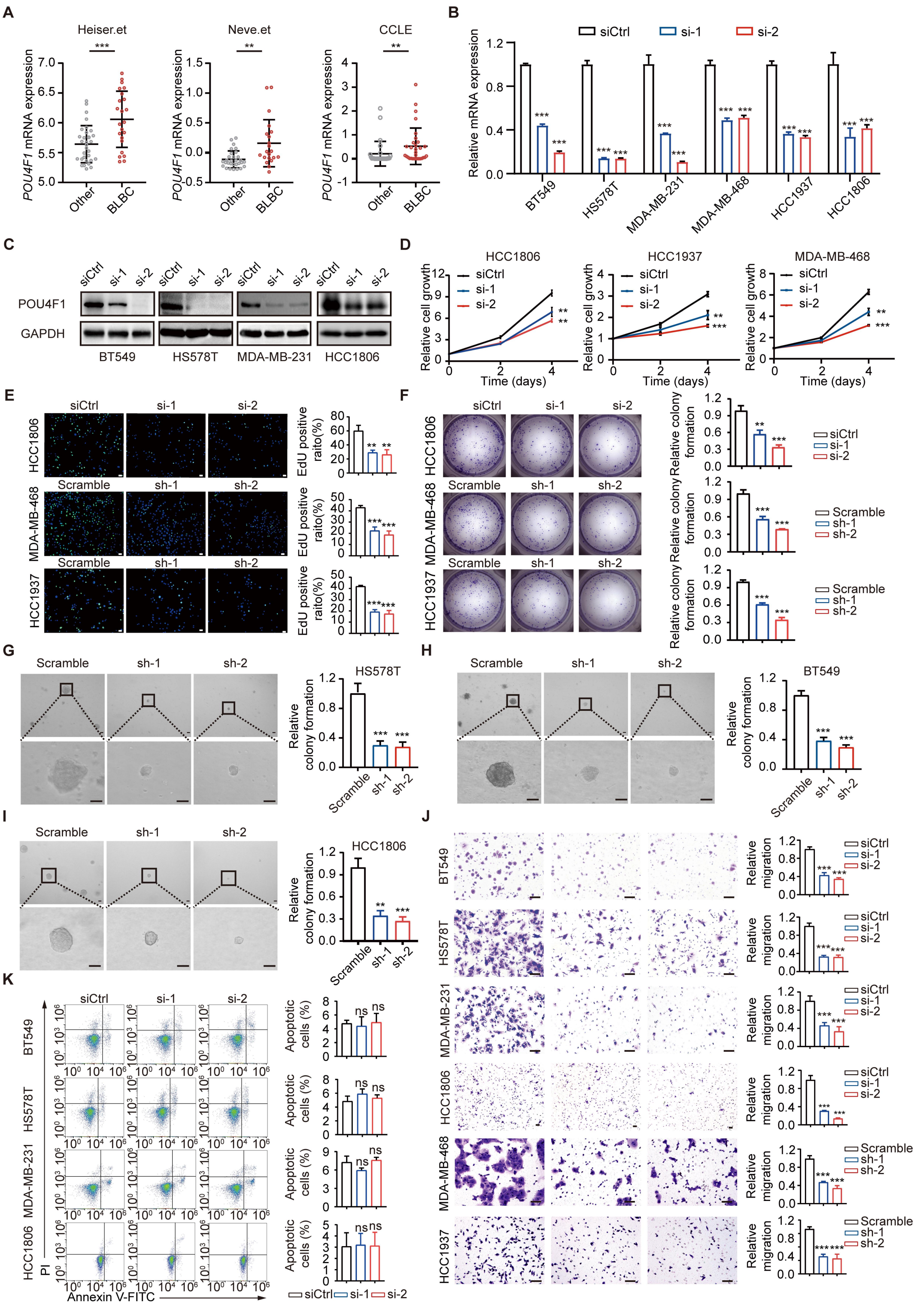

Figure S2. POU4F1 knockdown influence cell proliferation, colony formation and migration but not apoptosis of BLBC in vitro. A) *POU4F1* mRNA expression of BLBC and non-BLBC cell lines in Heiser (n = 54, BLBC = 23, other = 31), Neve (n =

51, BLBC = 21, other = 30) and CCLE (n = 59, BLBC = 31, other = 28) datasets. B, C) The knocking-down efficiency of two independent siRNA in BLBC cell lines, as validated by qRT-PCR (B) and western blotting (C). D, E) Cell proliferation of HCC1806, HCC1937 and MDA-MB-468 with POU4F1 knockdown, detected by CCK8 cell proliferation assay (D) and 5-Ethynyl-2'-deoxyuridine (EdU) assay (E). Scale bar, 50 μm. F) Representative images and quantitation of the colony formation of HCC1806, MDA-MB-468 and HCC1937 with POU4F1 knockdown. G-I) Representative images and quantitation of the adherent-independent colony formation in soft agar of HS578T (G), BT549 (H) and HCC1806 (I) after shRNA-mediated knockdown of POU4F1. Scale bar, 50 μm. J) Representative images and quantitation of cell migration of BLBC cell lines with POU4F1 knockdown, assessed through Boyden chambers assay. Scale bar, 50 μm. K) The proportion of apoptotic cells in 4 BLBC cell lines 36 h after siRNA-mediated knockdown of POU4F1, assessed by A nnexin V/ PI staining and flow cytometry. Data were presented as mean ± S.D. n = 3. ns, no significance, $^{**}P < 0.01$, $^{***}P < 0.001$ compared with other by two-tailed Student's t-test (A), compared with siCtrl or scramble by two-tailed one-way ANOVA and Dunnett's multiple-comparisons test (B, D-K).

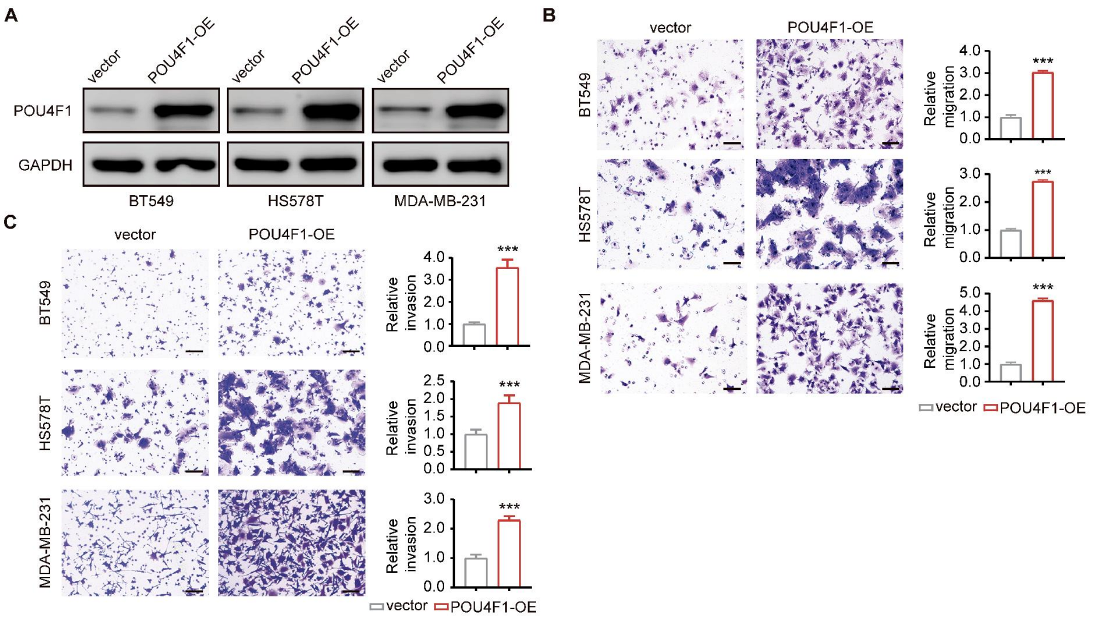

Figure S3. Overexpressing POU4F1 enhanced cell migration and invasion of BLBC in vitro. A) Western blotting showing POU4F1 expression in POU4F1-overexpressing BLBC cell lines. B, C) Representative images and quantitation of cell migration (B) and invasion (C) of POU4F1-overexpressing BLBC cell lines. Scale bar, 50 μm. Data were presented as mean ± S.D. $n = 3$. ***$P < 0.001$ compared with vector by two-tailed Student's t-test.

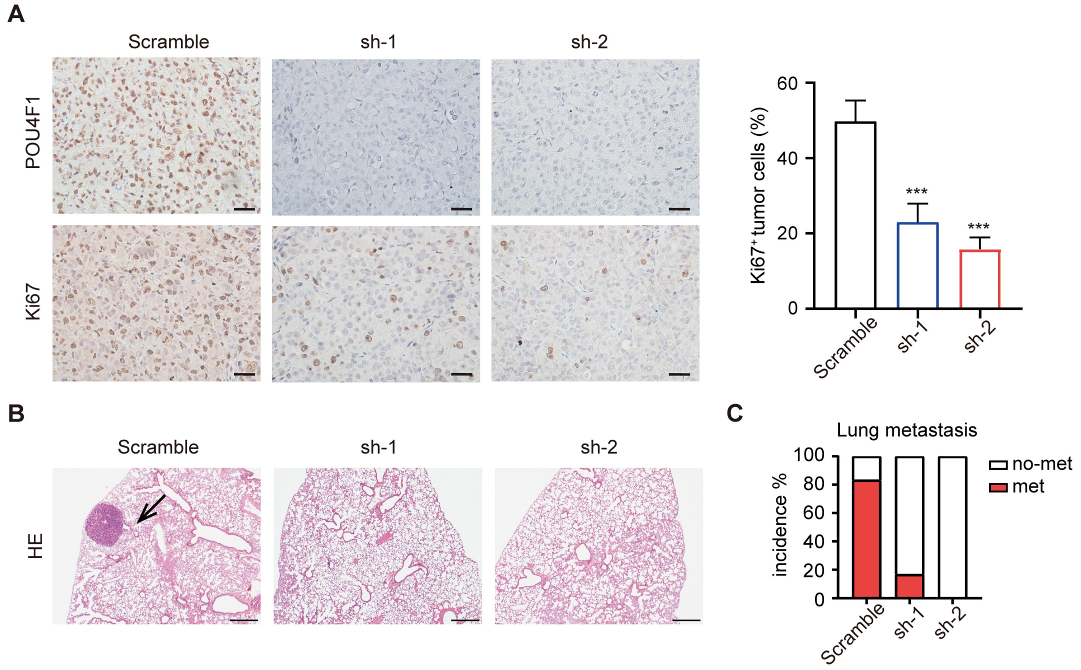

Figure S4. Knocking down of POU4F1 decreases cell proliferation and lung metastasis in vivo. A) Representative IHC staining images and quantification of Ki67 positive tumor cells in xenografts of MDA-MB-231 from each group. Data were presented as mean ± S.D., n = 6. ***P < 0.001 by two-tailed one-way ANOVA and Dunnett's multiple-comparisons test. Scale bar, 50 μ m. B) Representative H&E staining images of lung sections from each group. Arrow indicated lung metastasis nodule, n = 6. Scale bar, 500 μm. C) The incidence of lung metastasis of xenografts mice from each group (n = 6), assessed by H&E staining of lung sections.

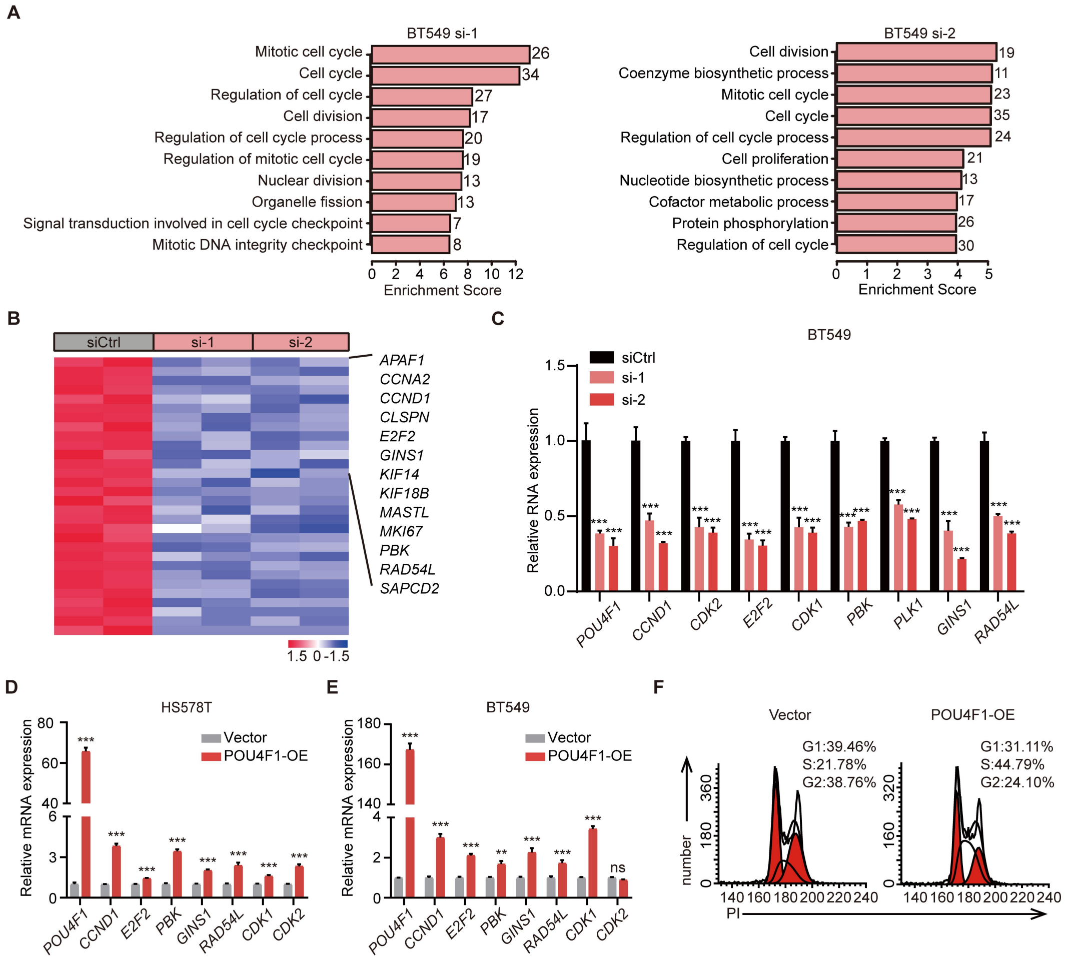

Figure S5. POU4F1 regulates cell cycle progression in BLBC. A) Top 10 GO categories of the differentially downregulated genes (fold change > 1.5 and $P < 0.05$) in BT549 transfected with POU4F1 siRNAs and control siRNA, as measured by RNA-sequencing with two biological duplicates. B) Heatmap of the overlap differentially downregulated genes of two siRNA targeting POU4F1 in BT549 cells. C-E) qRT-PCR detection of the expression of the representative cell cycle-related genes in HS578T and BT549 cells with POU4F1 knockdown and overexpression. F) Representative flow cytometry histograms and quantification of cell cycle distribution in POU4F1-overexpressing HS578T. Data were presented as mean ± S.D. (C-E). n = 3. ns, no significance, $^{**}P < 0.01$, $^{***}P < 0.001$ compared with siCtrl (C) or vector

(D, E) by two-tailed Student's t-test (D, E) and two-tailed one-way ANOVA and Dunnett's multiple-comparisons test (C).

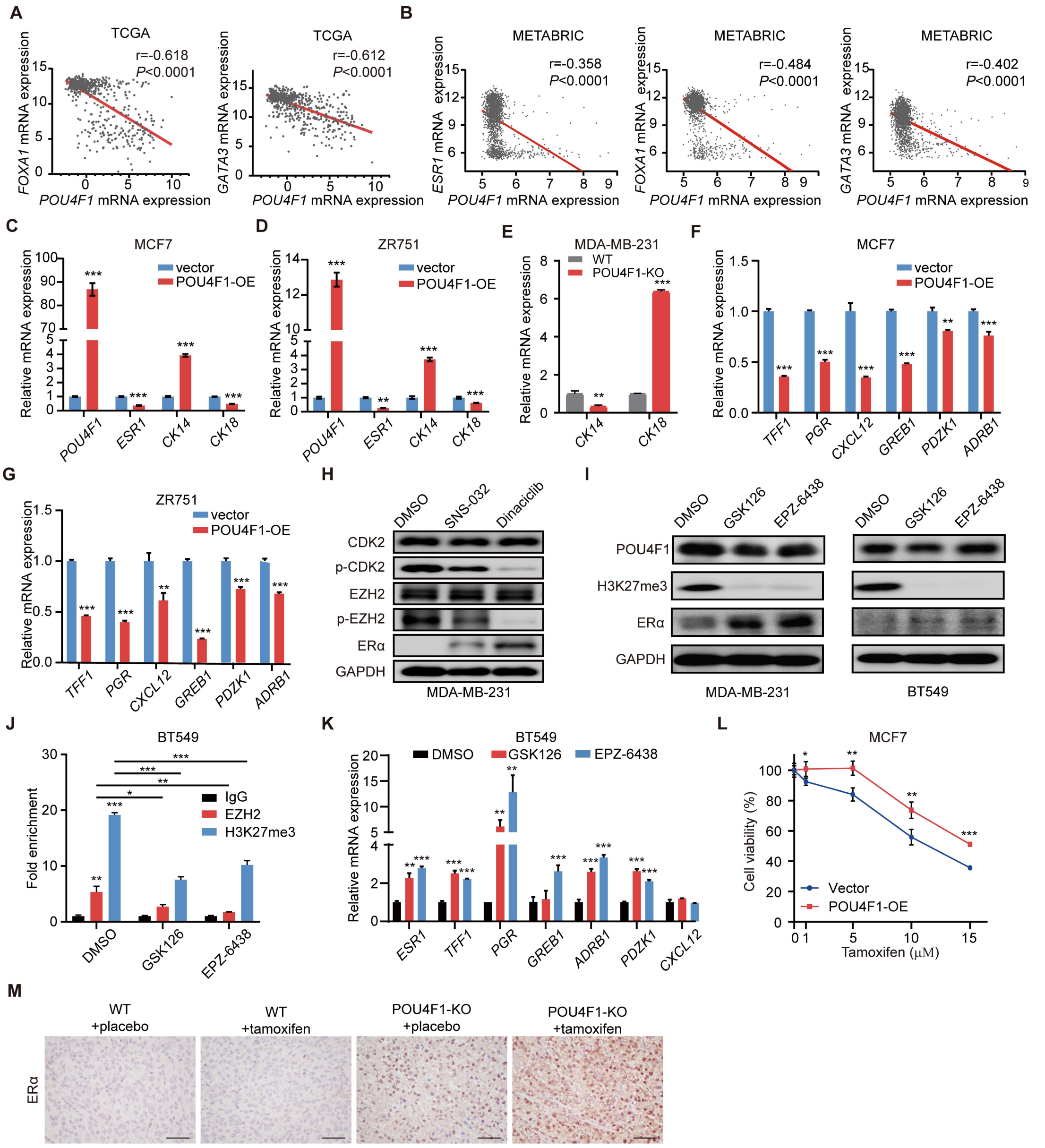

Figure S6. Targeting POU4F1 predisposes breast cancer cells to the conversion between luminal-like and basal-like phenotypes through CDK2/EZH2 axis. A, B) Scatter plots showing the Pearson correlation between POU4F1 and FOXA1, GATA3 in breast cancer patients of the TCGA (A) and METABRIC cohorts (B). Pearson's correlation coefficient r and two-tailed *P* value were shown. C, D) The mRNA expression of *ESR1*, basal marker *CK14* and luminal marker *CK18* in vector/POU4F1-overexpressing MCF7 and ZR751 cells were detected by qRT-PCR.

E) The mRNA expression of *CK14* and *CK18* in WT/POU4F1-KO MDA-MB-231 cells were detected by qRT-PCR. F, G) The mRNA expression of the downstream genes of ERα in POU4F1-overexpressing MCF7 and ZR751 were detected by qRT-PCR. H-I) Western blotting showing indicated protein expression in MDA-MB-231 cells treated with DMSO, 100nM SNS-032 and 25nM Dinaciclib for 72h (H) and MDA-MB-231 and BT549 cells treated with DMSO, 5 μM GSK126 and 10 μM EPZ-6438 for 72h (I). J-K) ChIP-qPCR analysis of the enrichment of IgG, EZH2 and H3K27me3 at the promoter of *ESR1* (J) and qRT-PCR analysis of the expression of *ESR1* and its downstream genes (K) in BT549 cells treated with DMSO, 5 μM GSK126 and 10 μM EPZ-6438 for 72h. L) Cell viability of POU4F1-overexpressing MCF7 cells treated with increasing concentrations of 4-hydroxytamoxifen for 72h. M) Representative IHC staining images of ERα in MDA-MB-231 xenografts from each group. Scale bar, 50 μm. Data were presented as mean ± S.D., $n = 3$ (C-G, J-L). *P < 0.05, **P < 0.01, ***P < 0.001 compared with vector (C, D, F, G, L), WT (E) and DMSO (J, K) by two-tailed Student's t-test (C-G, L) and two-tailed one-way ANOVA and Bonferroni (J) or Dunnett's (K) multiple-comparisons test.

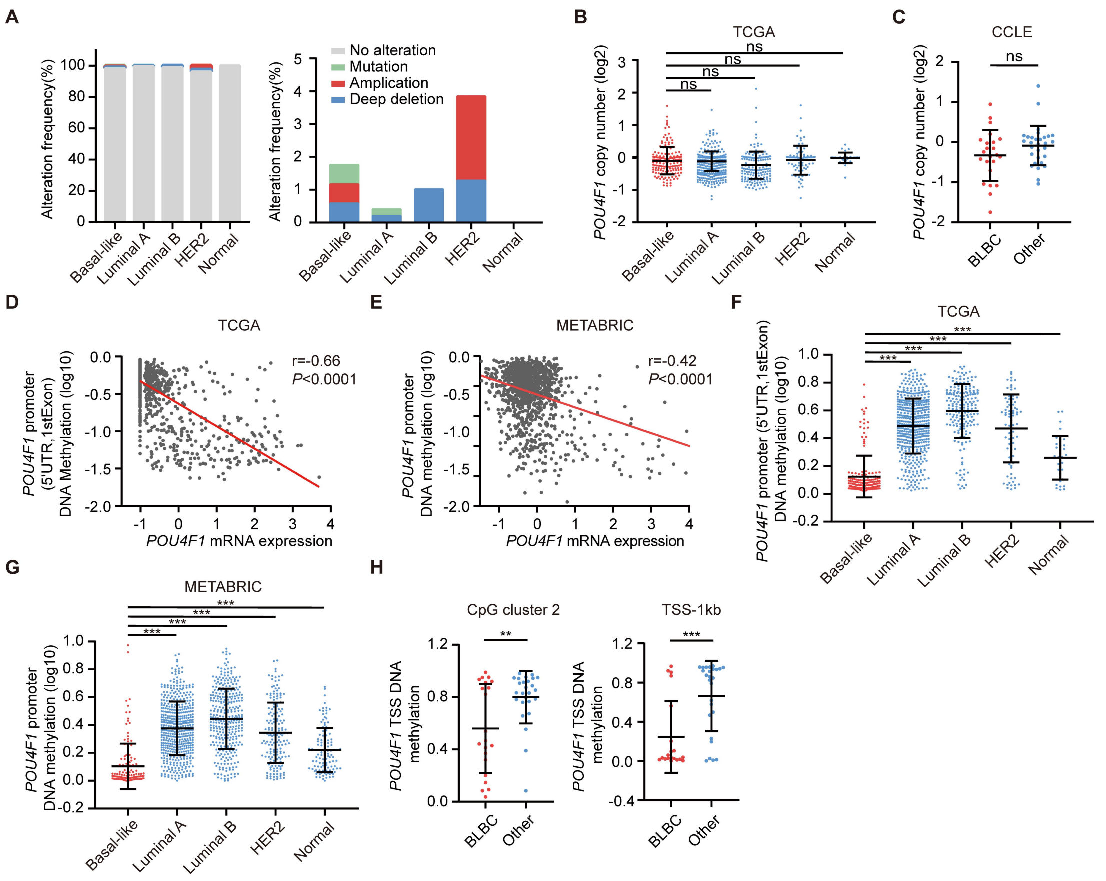

Figure S7. High expression of POU4F1 in BLBC is associated with DNA hypomethylation. A) Gene alteration frequencies and types of *POU4F1* in different subtypes of breast cancer in the TCGA cohort (n = 981). B) Copy number values of *POU4F1* across different subtypes of breast cancer in the TCGA cohort (n = 981). C) Copy number values of *POU4F1* in BLBC (n = 31) and non-BLBC (n = 28) cell lines in the CCLE project. D, E) Scatter plots showing the Pearson correlation between *POU4F1* mRNA expression (z-scores) and its DNA methylation ($\log_{10}$) in breast cancer patients in the TCGA (n = 966) and METABRIC (n = 1271) cohorts. Pearson's correlation coefficient r and two-tailed P value were shown. F, G) DNA methylation β values in the *POU4F1* promoter across different subtypes of breast cancer patients using available WGBS data in the TCGA (Basal-like: n = 168, HER2-enriched: n = 78,

Luminal A: *n* = 488, Luminal B: *n* = 197, Normal-like: *n* = 35) and METABRIC (Basal-like: *n* =143, HER2-enriched: *n* = 174, Luminal A: *n* = 500, Luminal B: *n* = 339, Normal-like: *n* = 115) cohorts. H) DNA methylation β values of CpG clusters located in the *POU4F1* TSS in breast cancer cell lines from the CCLE project (BLBC: *n* = 21, other subtypes: *n* = 26). Data were presented as mean ± S.D. (B, C, F-H). ns, no significance, $^{**}P < 0.01$, $^{***}P < 0.001$ compared with BLBC by two-tailed Student's t-test (C, H) and two-tailed one-way ANOVA and Dunnett's multiple-comparisons test (B, F, G).

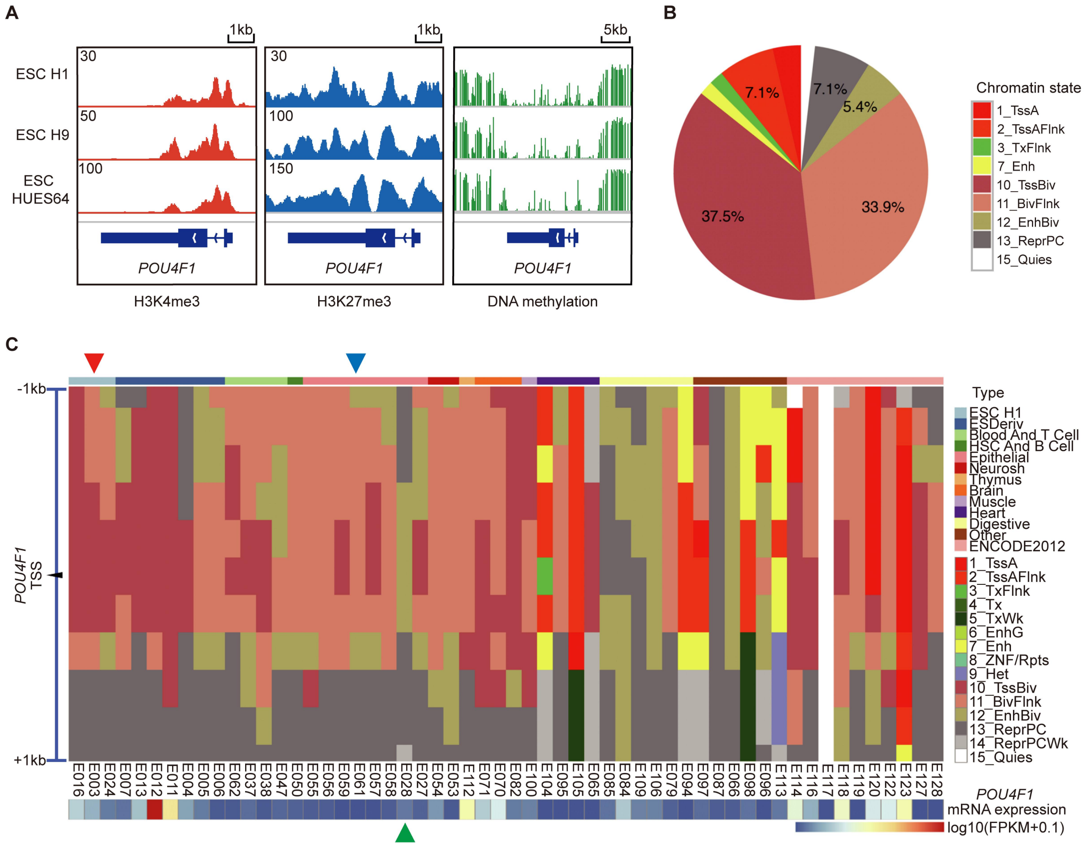

Figure S8. *POU4F1* gene locus maintains in a bivalent chromatin state in ESC and breast epithelium. A) ChIP-seq peak signals of H3K4me3, H3K27me3 and DNA methylation profiles in *POU4F1* gene locus of embryonic stem cells (ESC H1, HSC H9, HSC HUES64). The scale was shown in the upper left of each track. B) Pie chart showing the proportion of different chromatin states in the *POU4F1* TSS ± 1000 bp region across human normal cell types from the Epigenome Roadmap project. TssA, active TSS; TssAFlnk, flanking active TSS; TxFlnk, transcribed state at the 5′ and 3′ end of genes; Enh, enhancers; TssBiv, bivalent TSS; BivFlnk, flanking bivalent TSS/Enhancer; EnhBiv, bivalent enhancer; ReprPC, repressed PolyComb; Quies, quiescent/Low. C) The chromatin states of the *POU4F1* TSS ± 1000 bp region across human normal cell types form the Epigenome Roadmap project. Each column

represented a cell type. The upper track indicated tissue types. The mRNA expression of *POU4F1* of each cell type were shown by color in the bottom track. The red, blue and green triangles indicated embryonic stem cells, epithelial tissue and breast epithelium respectively. Tx, strong transcription; TxWk, weak transcription; EnhG, genic enhancers; ZNF/Rpts, ZNFgenes & repeats; Het, heterochromatin; ReprPCWk, weak repressed PolyComb.

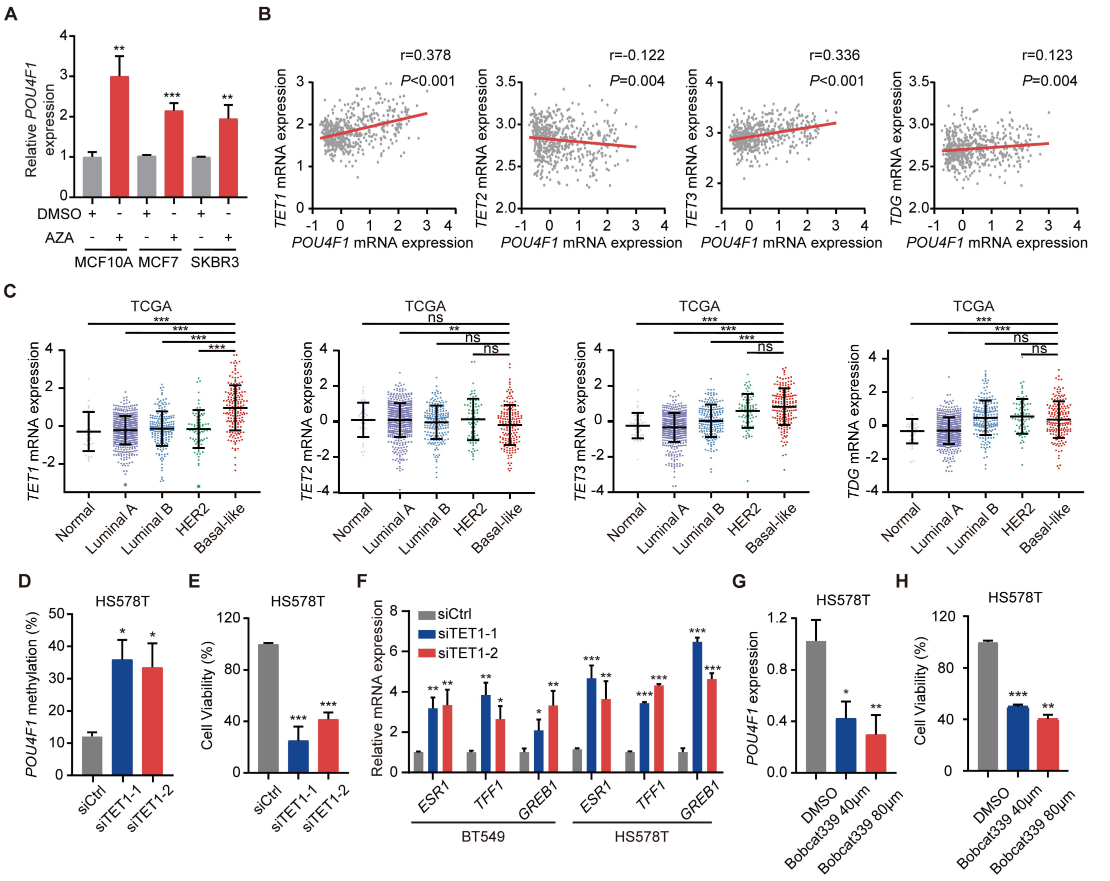

Figure S9. TET1 maintains DNA hypomethylation of POU4F1 and contributes to POU4F1 activation in BLBC. A) The mRNA expression of *POU4F1* in MCF10A, MCF7 and SKBR3 treated with 5-aza-2'-deoxycytidine (5-Aza-dC) for 72 h. B) Scatter plots showing the Pearson correlation between POU4F1 and TET1, TET2, TET3 and TDG in breast cancer patients of the TCGA cohort. Pearson's correlation coefficient r and two-tailed *P* value were shown. C) The mRNA expression of *TET1*, *TET2*, *TET3* and *TDG* across different subtypes of breast cancer patients in the TCGA cohort (Basal-like: *n* = 168, HER2-enriched: *n* = 78, Luminal A: *n* = 488, Luminal B: *n* = 197, Normal-like: *n* = 35). D) DNA methylation level of the CpG island in *POU4F1* promotor was analyzed by qMSP in HS578T transfected with TET1 siRNA.

E) Cell viability in HS578T transfected with TET1 siRNA. F) The mRNA expression of *ESR1* and its downstream genes in BT549 and HS578T transfected with TET1 siRNA was analyzed by qRT-PCR. G, H) *POU4F1* mRNA expression and cell viability in HS578T treated with indicated concentrations of Bobcat339 for 3 days. Data were presented as mean ± S.D., n = 3 (A, D-H). ns, no significance, $^{*}P < 0.05$, $^{**}P < 0.01$, $^{***}P < 0.001$ compared with DMSO (A, G, H), BLBC (C) and siCtrl (D-F) by two-tailed one-way ANOVA and Dunnett's multiple-comparisons test (C-H) and by two-tailed Student's t-test (A).

Supplementary Tables

Table S1. Correlation of POU4F1 expression in tumor slices with clinicopathologic characteristics in 107 cases of TNBC[a] patients from SYSMH

Characteristics	No. of patients	POU4F1 expression		*P* value
		Low n=81	High n=26	
**Age (y)**				
≤ 50	49	37 (75.51%)	12 (24.49%)	0.966
> 50	58	44 (75.86%)	14 (24.14%)	
**Menopause**				
Yes	52	38 (73.08%)	14 (26.92%)	0.538
No	55	43 (78.18%)	12 (21.82%)	
**Tumor size (cm)**				
≤ 2	47	42 (89.36%)	5 (10.64%)	0.007*
> 2	60	39 (65.00%)	21 (35.00%)	
**Histological grade**				
DCIS[b]-II	19	16 (84.21%)	3 (15.79%)	0.510
III	88	65 (73.86%)	23 (26.14%)	
**TNM stage**				
Tis[c]-I	42	39 (92.86%)	3 (7.14%)	0.002*
II-III	65	42 (64.62%)	23 (35.38%)	
**Lymph node metastasis**				
Yes	32	21 (65.63%)	11 (34.36%)	0.112
No	75	60 (80.00%)	15 (20.00%)	
**Ki67**				
≤ 30%	21	17 (80.95%)	4 (19.05%)	0.732
> 30%	86	64 (74.42%)	22 (25.58%)	

Abbreviations: [a)] TNBC: triple-negative breast cancer; [b)] DCIS: ductal carcinoma in situ; [c)] Tis: tumor in situ.* *P* <0.05, statistically significant. *P* values were determined by chi-squared test.

Table S2. Cox regression analysis of disease-free survival (DFS) in 107 cases of TNBC patients from SYSMH.

Characteristics	Univariate analysis			Multivariate analysis		
	HR[a]	95% CI[b]	*P* value	HR	95% CI	*P* value
**Age (≤50 vs. >50 years)**	2.403	0.636-9.077	0.196	-	-	-
**Menopause (Yes vs. No)**	3.029	0.802-11.445	0.102	-	-	-
**Tumor size (≤ 2 cm vs. > 2 cm)**	1.512	1.151-1.985	0.003*	1.389	1.024-1.883	0.035*
**Histological grade (DCIS-II vs. III)**	1.046	0.225-4.855	0.954	-	-	-
**TNM stage (Tis-I vs. II-III)**	3.562	0.766-16.570	0.105	-	-	-
**Lymph node status (Yes vs. No)**	4.791	1.398-16.417	0.013*	-	-	0.122
**Ki67 (≤ 30% vs. > 30%)**	0.749	0.196-2.857	0.672	-`	-	-
**POU4F1 expression (as continuous)**	1.292	1.077-1.550	0.006*	1.245	1.030-1.505	0.023*

Abbreviations: a) HR: hazard ratio; b) CI: confidence interval.* *P* <0.05, statistically significant. *P* values were determined by Cox regression analysis.

Table S4. siRNA, shRNA and sgRNA sequence

Target name	Sequence
siPOU4F1-1/shPOU4F1-1	5’-CCACGTACCACACGATGAA-3’
siPOU4F1-2/shPOU4F1-2	5’-CCGAGAAACTGGACCTCAA-3’
siTET1-1	5’-GCCCACAGTTGTAAGTTAT-3’
siTET1-2	5’-CCCACCTCCAGTCTTAATA-3’
siCtrl/scramble	5’-CCTAAGGTTAAGTCGCCCTCG-3’
sgPOU4F1	5’-GATGTGGTCCAGCAGATCGC-3’
sgNC	5’-CCCGAATCTCTATCGTGCGG-3’

Table S5. Sequence of primers used in the qRT-PCR for indicated genes.

Gene	Forward	Reverse
ACTB	5'-CATGTACGTTGCTATCCAGGC-3'	5'-CTCCTTAATGTCACGCACGAT-3'
ADRB1	5'-ATCGAGACCCTGTGTGTCATT-3'	5'-GTAGAAGGAGACTACGGACGAG-3'
CCND1	5'-GCTGCGAAGTGGAAACCATC-3'	5'-CCTCCTTCTGCACACATTTGAA-3'
CDK1	5'-AAACTACAGGTCAAGTGGTAGCC-3'	5'-TCCTGCATAAGCACATCCTGA-3'
CDK2	5'-AATCCGCCTGGACACTGAGACT-3'	5'-GTGGAGGACCCGATGAGAATGG-3'
CK14	5'-GGACCTGAGCCGCATTCTGAAC-3'	5'-TCCTCCACGCTGCCAATCATCT-3'
CK18	5'-CGCTCCACCTTCTCCACCAACTA-3'	5'-TTCTCGGTCTCCAGGCTCCTCA-3'
CXCL12	5'-ATTCTCAACACTCCAAACTGTGC-3'	5'-ACTTTAGCTTCGGGTCAATGC-3'
E2F2	5'-GCTGTCCAGTCTGCTAGAATGCT-3'	5'-TGCCTCTGCCTACCAAGTCCTT-3'
ESR1	5'-CTCTCCCACATCAGGCACA-3'	5'-CTTTGGTCCGTCTCCTCCA-3'
EZH2	5'-GGACCACAGTGTTACCAGCAT-3'	5'-GTGGGGTCTTTATCCGCTCAG-3'
GAPDH	5'-ACAACTTTGGTATCGTGGAAGG-3'	5'-GCCATCACGCCACAGTTTC-3'
GINS1	5'-ACGAGGATGGACTCAGACAAG-3'	5'-TGCAGCGTCGATTTCTTAACA-3'
GREB1	5'-ATGGGAAATTCTTACGCTGGAC-3'	5'-CACTCGGCTACCACCTTCT-3'
PBK	5'-TACTATGCAGCGTTGGGAACT-3'	5'-GCAGAAGGACGATCTTTAGGGTC-3'
PDZK1	5'-GAACTGCCCAATGGCTCTGT-3'	5'-CCCCGAATCGCATTTAAGTGAA-3'
PGR	5'-TGCTATGTGCTGGAGGCTTCTG-3'	5'-TGGTCAGTTGGAGGCAGGCATA-3'
PLK1	5'-CCTGCACCGAAACCGAGTTAT-3'	5'-CCGTCATATTCGACTTTGGTTGC-3'
POU4F1	5'-GGGCAAGAGCCATCCTTTCAA -3'	5'-CTGTTCATCGTGTGGTACGTG -3'
RAD54L	5'-TTTACGCCAGAGTCCAGAGTG-3'	5'-ATGAAGGCGGAAGGTCTCATA-3'
TET1	5'- CGCTACGAAGCACCTCTCTTA-3'	5'- CTTGCATTGGAACCGAATCATTT-3'
TFF1	5'-CCCTCCCAGTGTGCAAATAAG-3'	5'-GAACGGTGTCGTCGAAACAG-3'

Table S6. Sequence of primers used in the ChIP-qPCR.

Name		Sequence
POU4F1 promoter Primer 1	Forward	5’-AACGTGGGTTTGTACACGGT-3’
	Reverse	5’-CACAAGGAGCGTGTGCTTTC-3’
CDK2 promoter Primer 1	Forward	5’-CCGGGCAAGAGCTAACTGAA-3’
	Reverse	5’-CTGCCTCATAGCATACCCCG-3’
CDK2 promoter Primer 2	Forward	5’-AAATGAGTGGGAGACGCCTG-3’
	Reverse	5’-ATAGGTGCTTTGCTGGCTGT-3’
CCND1 promoter Primer 1	Forward	5’-AACCTTCGGTGGTCTTGTCC-3’
	Reverse	5’-TACCTTGACCAGTCGGTCCT-3’
CCND1 promoter Primer 2	Forward	5’-GCCGGAATGAAACTTGCACA-3’
	Reverse	5’-CCATATCCAAGCCGGCAGAA-3’
ESR1 promoter Primer 1	Forward	5’-GTCTGCCAGTAGGCAGTTGA-3’
	Reverse	5’-ATACCCACTCCTGTCAGTGC-3’
ESR1 promoter Primer 2	Forward	5’-GGGGGTTGATGAAAACATGGG-3’
	Reverse	5’-CTTCCCCCAGCAAGTCAAGA-3’

Table S7. Sequence of primers used in the quantitative methylation-specific PCR.

Name		Sequence
POU4F1 methylated	Forward	5'- GTTATTATAAGAGCGGTTTTTACGC-3'
	Reverse	5'- GCTAACAAACATCAACTATCTCCGT-3'
POU4F1 unmethylated	Forward	5'- TTATTATAAGAGTGGTTTTTATGTGT-3'
	Reverse	5'- ACTAACAAACATCAACTATCTCCATC-3'
